# Tryptophan Operon Diversity Reveals Evolutionary Trends among Geographically Disparate Chlamydia trachomatis Ocular and Urogenital Strains Affecting Tryptophan Repressor and Synthase Function

**DOI:** 10.1128/mBio.00605-21

**Published:** 2021-05-11

**Authors:** Sankhya Bommana, Naraporn Somboonna, Gracie Richards, Maryam Tarazkar, Deborah Dean

**Affiliations:** a Department of Pediatrics, University of California San Francisco, Oakland, California, USA; b Department of Bioengineering, Joint Graduate Program, University of California San Francisco and University of California Berkeley, San Francisco, California, USA; c Department of Medicine, University of California San Francisco, San Francisco, California, USA; d Bixby Center for Global Reproductive Health, University of California San Francisco, San Francisco, California, USA; Fred Hutchinson Cancer Research Center

**Keywords:** *Chlamydia trachomatis*, evolution, phylogeny, protein modeling and docking, sexually transmitted infections, tryptophan biosynthesis, tryptophan operon

## Abstract

The obligate intracellular pathogen Chlamydia trachomatis (*Ct*) is the leading cause of bacterial sexually transmitted infections and blindness globally. To date, *Ct* urogenital strains are considered tryptophan prototrophs, utilizing indole for tryptophan synthesis within a closed-conformation tetramer comprised of two α (TrpA)- and two β (TrpB)-subunits. In contrast, ocular strains are auxotrophs due to mutations in TrpA, relying on host tryptophan pools for survival. It has been speculated that there is strong selective pressure for urogenital strains to maintain a functional operon. Here, we performed genetic, phylogenetic, and novel functional modeling analyses of 595 geographically diverse *Ct* ocular, urethral, vaginal, and rectal strains with complete operon sequences. We found that ocular and urogenital, but not lymphogranuloma venereum, TrpA-coding sequences were under positive selection. However, vaginal and urethral strains exhibited greater nucleotide diversity and a higher ratio of nonsynonymous to synonymous substitutions [Pi(a)/Pi(s)] than ocular strains, suggesting a more rapid evolution of beneficial mutations. We also identified nonsynonymous amino acid changes for an ocular isolate with a urogenital backbone in the intergenic region between TrpR and TrpB at the exact binding site for YtgR—the only known iron-dependent transcription factor in *Chlamydia*—indicating that selective pressure has disabled the response to fluctuating iron levels. *In silico* effects on protein stability, ligand-binding affinity, and tryptophan repressor (TrpR) affinity for single-stranded DNA (ssDNA) measured by calculating free energy changes (ΔΔ*G*) between *Ct* reference and mutant tryptophan operon proteins were also analyzed. We found that tryptophan synthase function was likely suboptimal compared to other bacterial tryptophan prototrophs and that a diversity of urogenital strain mutations rendered the synthase nonfunctional or inefficient. The novel mutations identified here affected active sites in an orthosteric manner but also hindered α- and β-subunit allosteric interactions from distant sites, reducing efficiency of the tryptophan synthase. Importantly, strains with mutant proteins were inclined toward energy conservation by exhibiting an altered affinity for their respective ligands compared to reference strains, indicating greater fitness. This is not surprising as l-tryptophan is one of the most energetically costly amino acids to synthesize. Mutations in the tryptophan repressor gene (*trp*R) among urogenital strains were similarly detrimental to function. Our findings indicate that urogenital strains are evolving more rapidly than previously recognized with mutations that impact tryptophan operon function in a manner that is energetically beneficial, providing a novel host-pathogen evolutionary mechanism for intracellular survival.

## INTRODUCTION

The obligate intracellular pathogen Chlamydia trachomatis (*Ct*) is the leading cause of bacterial sexually transmitted infections (STIs) and preventable blindness worldwide (1). Over 130 million cases of *Ct* STIs occur annually and can lead to severe reproductive complications such as chronic pelvic pain, tubal factor infertility, ectopic pregnancy, and preterm birth (2). *Ct* is also responsible for lymphogranuloma venereum (LGV), an invasive disease that can cause hemorrhagic proctitis and spread via regional lymphatics to the inguinal lymph nodes, forming buboes with subsequent suppuration (2). LGV is endemic in global tropical and subtropical regions as well as worldwide among men who have sex with men (3, 4). An estimated 232 million people are at risk of blindness from trachoma, a chronic ocular disease that occurs primarily in endemic tropical developing countries (5). Repeat and/or persistent infection can induce an immunopathogenic response that can lead to progression from conjunctival inflammation to tissue fibrosis, scarring, and blindness (6).

Molecular typing of *Ct omp*A, referred to as *omp*A genotyping, has been used to understand the tissue tropism and molecular epidemiology of *Ct* infections (7). In general, ocular *omp*A genotypes A, B, Ba, and C cause trachoma; urogenital genotypes D, Da, E, F, G, H, I, Ia, J, Ja, and K are linked to urogenital infections while only some of these genotypes cause rectal infections; and genotypes L1, L2, L2a, L2b, L2c, and L3 are responsible for LGV (6). The non-LGV strains are referred to as the trachoma noninvasive biological variant (biovar) while LGV strains are considered the invasive biovar (6).

With the advent of multilocus sequence typing (MLST) and partial and whole-genome sequencing (WGS), there has been a greater understanding of strain diversity, strain emergence from recombination (e.g., Da/ocular, B/urogenital, L2/D, Ja/E recombinant strains), and tissue tropism (8–17). We now know that there are *omp*A genotypes B, Ba, and C that are actually urogenital strains because they have a urogenital strain backbone (18, 28). Genes involved in apparent tissue tropism include *omp*A, *tar*P, *inc*A, toxin-like genes, and *trp*A, among others (19).

*Ct* relies heavily on host nutrient supplies and biochemical processes to sufficiently proliferate within the host cell (20). Although urogenital strains are tryptophan prototrophs, auxotrophic ocular strains depend on the availability of host tryptophan pools for survival. Tryptophan synthesis is energetically costly, and, therefore, *Ct* ocular strains and other intracellular bacteria have evolved to scavenge this essential metabolite from their hosts (21). Urogenital strains are able to synthesize tryptophan from substrates such as indole, when tryptophan is scarce, using their intact functional tryptophan synthase (TS). Tryptophan scarcity occurs when there is competition for this amino acid (aa) from other microbes in the respective microbiome and as a result of the tryptophan catabolism by indoleamine 2,3-dioxygenase 1 (IDO1). IDO1 is induced by interferon gamma (IFN-γ), an innate immune cytokine that is stimulated in response to *Ct* infection (22). An intact operon is thought, therefore, to enable *Ct* to escape immune surveillance and survive.

The operon consists of the tryptophan repressor, TrpR, and two β (TrpB)- and two α (TrpA)-subunits that form an αββα tetrad with an intramolecular tunnel. In the tunnel, indole is bound and converted to l-tryptophan (23, 24). Ocular strains, in contrast to urogenital strains, have lost this function due to a single nucleotide deletion and frameshift in *trp*A that encodes a truncated TrpA (25, 26). Initial studies revealed high homology among urogenital and ocular strains for the respective operon sequence (25). More recently, we discovered that long-term persistence of a urogenital clinical F strain occurred due to a novel mutation in *trp*A resulting in elongation of TrpA by 2 aa with an altered αββα structure and markedly decreased TS with lower uptake of tryptophan for metabolism compared to the reference strain (26). While it is thought that there is strong selective pressure for urogenital strains as opposed to ocular strains to maintain a functional operon (25), our recent data indicate that this may not be entirely true.

**FIG 1:**
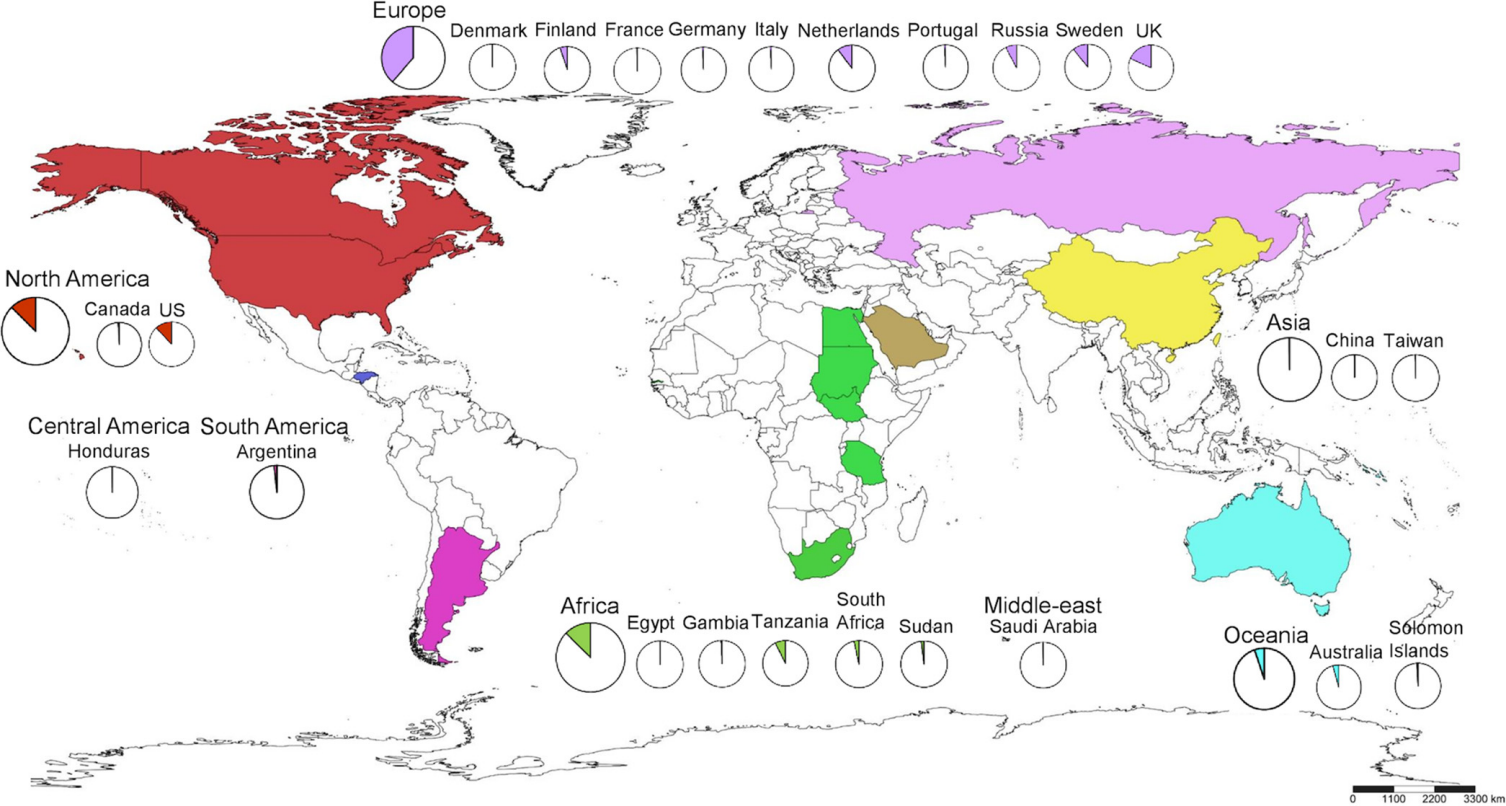
Global distribution of Chlamydia trachomatis strains harboring the tryptophan operon used in this study. Colors indicate the geographic areas or continents that were the sources of the samples as shown in the key. Circles within countries show the number of samples from that country. Countries neither sampled nor included in this study are shown in white. The scale bar underneath the map provides a visual indication of the size of features, and distance between features, on the map. Map was generated by SimpleMappr.

Current studies of tryptophan operons are based on *Ct* strains from Tanzania, Gambia, the United States, and Canada, often with a focus on TrpA and TrpB. We sought to expand this work by examining the entire tryptophan operon from 595 genomes representing ocular, urethral, vaginal, rectal, and LGV strains from 24 countries and all continents of the world except for Antarctica (Fig. 1). Here, we performed genetic, phylogenetic, and novel functional protein modeling analyses on these geographically diverse *Ct* strains to elucidate the evolution of the operon and its changing role in tissue tropism and disease pathogenesis.

## RESULTS

### Tryptophan operon phylogeny revealed distinct LGV and trachoma linages with prevalent, nonprevalent, and mixed urogenital clades with greater diversity for *trp*A.

**FIG 2:**
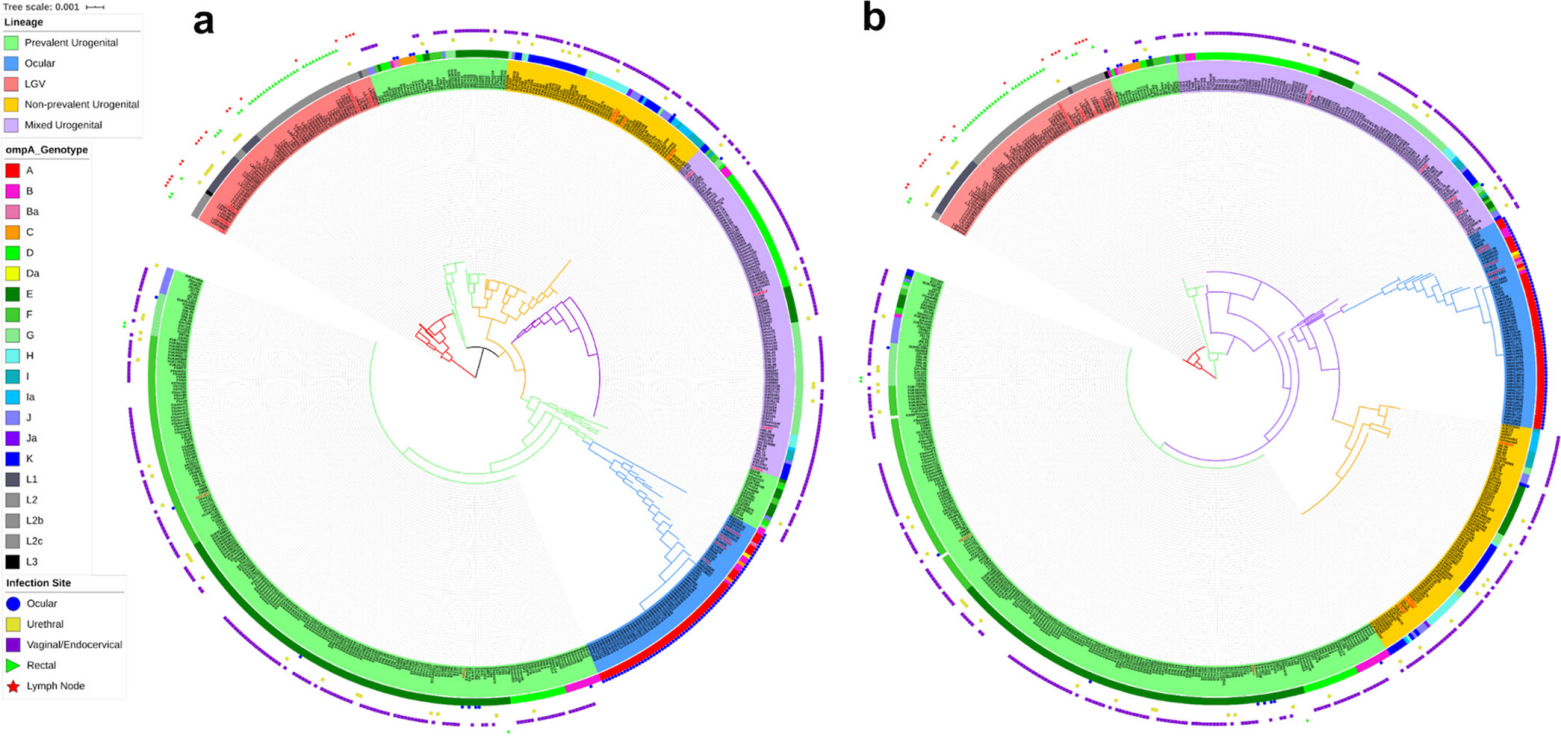
Interactive phylogenetic trees for *trp*RBA (a) (see https://itol.embl.de/tree/209360135466881582240570) and *trp*A (b) (see https://itol.embl.de/tree/7616769102203851589927158) depicting *Ct* lineages: LGV, nonprevalent and prevalent urogenital, mixed urogenital, and a more recent ocular subclade. The relationship between these lineages with their *omp*A genotype and site or infection are represented as inner and outer ring color strips and as a binary data set. The tree was constructed by FastTree with a generalized time-reversible model based on a MAFFT alignment of concatenated *trp*RBA and *trp*A sequences for each of the 595 strains in the data set (see Materials and Methods). Both were executed in Geneious. *omp*A and site-of-infection data of the strains together with the phylogenetic tree were used to generate this figure in iTOL.

Tryptophan operon sequences consisting of *trp*R, intergenic region (IGR), *trp*B, and *trp*A, in that order, were available for 595 clinical and reference samples; 12 ocular strains from Sudan (27) were excluded due to read quality (Fig. 1; see also Data Set S1 in the supplemental material). Phylogenetics revealed two distinct lineages representing the LGV and trachoma biovars. The latter branched into four distinct clades of prevalent, nonprevalent, and mixed urogenital strains with the ocular strains forming a more recent branch from an ancestral prevalent urogenital subclade (Fig. 2a, inner circle; https://itol.embl.de/tree/7616769102203851589927158). In general, the prevalent urogenital strains consisted of D, Da, E, and F with some J and Ja strains; nonprevalent strains included all others (11).

10.1128/mBio.00605-21.6 TABLE S1 Primary recombination analysis in the tryptophan operon for global C. trachomatis strains (*n* = 595) using RDP4. Download Table S1, PDF file, 0.08 MB. Copyright © 2021 Bommana et al. 2021 Bommana et al.

10.1128/mBio.00605-21.10 DATA SET S1 List of C. trachomatis strains used in this study and associated metadata for the tryptophan operon nucleotide sequences. Download Data Set S1, PDF file, 0.2 MB. Copyright © 2021 Bommana et al. 2021 Bommana et al.

The first branch of prevalent strains contained ocular *omp*A genotypes with urogenital backbones isolated from the eyes of Aboriginal children (18) and urogenital *omp*A genotypes with urogenital backgrounds isolated from the eyes of patients in Denmark and Argentina (Fig. 2a; blue dots at ∼11 o’clock; Table S1).

*trp*A phylogeny similarly contained both biovar lineages, although the earliest ancestor after LGV was the ocular China B_QH111L strain with a D/G urogenital backbone (28) (Fig. 2b, inner circle; https://itol.embl.de/tree/7616769102203851589927158). There was greater genetic diversity among nonprevalent urogenital and ocular branches compared to prevalent urogenital strains. Ocular A strains from Tanzania were a relatively recent clonal population compared to all other ocular strains with B_TZ1A828 as the ancestral strain (Fig. 2b). Overall, *trp*A phylogeny was somewhat congruent with operon phylogeny.

### Tryptophan operon analyses revealed no recombination but hot spots of genetic variation and evidence of signatures for LGV, ocular, and urogenital tissue tropism.

Using the MAFFT alignment and a series of programs run in RDP4, there was no evidence for recombination involving the operon (Tables S1 and S2).

10.1128/mBio.00605-21.7 TABLE S2 Secondary analysis of putative C. trachomatis recombinants involving the tryptophan operon identified by RDP4 using Recco. Download Table S2, PDF file, 0.05 MB. Copyright © 2021 Bommana et al. 2021 Bommana et al.

10.1128/mBio.00605-21.8 TABLE S3 Nucleotide polymorphisms in *trp*B for C. trachomatis clinical and reference ocular strains. Download Table S3, PDF file, 0.09 MB. Copyright © 2021 Bommana et al. 2021 Bommana et al.

Single nucleotide polymorphism (SNP) analysis of individual tryptophan operon coding sequences (CDS) was performed by segregating strains based on phylogenetic clustering with the reference strain sequences (Fig. S1) in addition to considering *omp*A genotype and anatomic origin.

10.1128/mBio.00605-21.1 FIG S1 Concatenated *trp*RBA phylogenetic tree of 21 *Ct* reference strains constructed by FastTree with a generalized time-reversible model based on a MAFFT alignment of *trp*RBA sequences (see Materials and Methods). Both were executed in Geneious (https://www.geneious.com). The tree scale indicates the distance between the sequences, and the branch length indicates the number of substitutions that have occurred in that branch. Download FIG S1, PDF file, 0.09 MB. Copyright © 2021 Bommana et al. 2021 Bommana et al.

**FIG 3:**
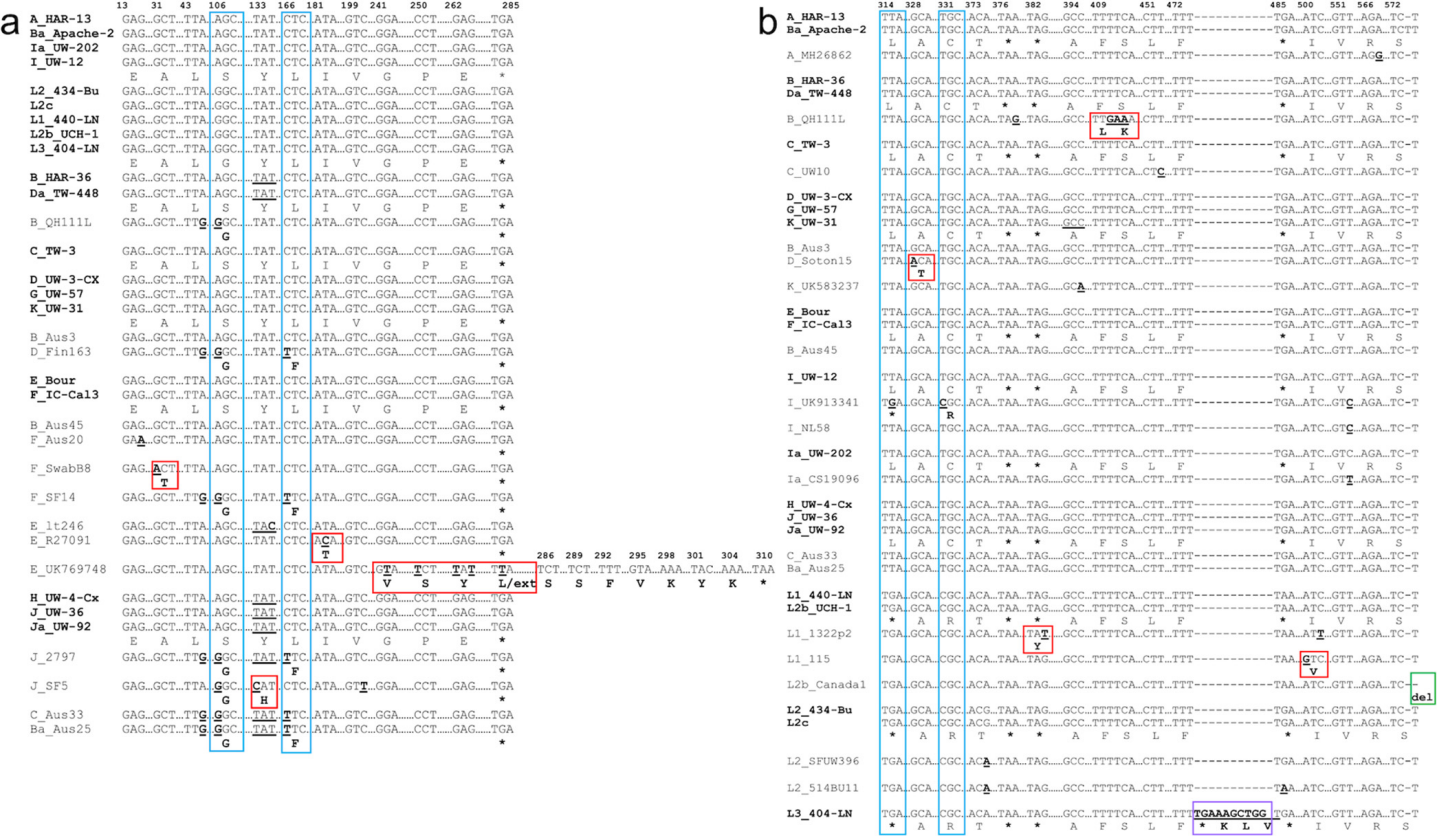
Partial nucleotide sequences of *trp*R (a) and IGR (b) with regions of single nucleotide polymorphism (SNPs) and indels in comparison to the 21 *Ct* reference strain sequences (in bold). Australian aboriginal strains Ba_Aus25, B_Aus3, B_Aus45, and C_Aus33 were included with the urogenital strains due to their “true” genome sequence identity as urogenital strains. Homologous regions in the sequence are not shown and are denoted as “… .” Dashes denote nucleotide deletion(s). Nucleotides in bold denote substitutions, and bold aa letters denote nonsynonymous aa substitutions compared to the reference strain. Nucleotide and/or aa changes that are unique to a single strain are boxed in red; changes common across several sequences/variants are boxed in blue. “ins,” insertion (boxed in purple); “fs,” frameshift; “ext,” extension; “del,” deletion (boxed in green).

*trp*R is considered highly conserved with only two common nucleotide (nt) positions of nonsynonymous substitutions confined to D, F, and J stains (Fig. 3a; blue boxes). Only reference strains and those with mutations are shown. All other strains were similar to reference or clinical strains. Unique nonsynonymous substitutions are also noted (Fig. 3a; red boxes). Strain E_UK769748 had four unique nonsynonymous substitutions that extended the protein by 7 aa (Fig. 3a; red box). The IGR had two common SNPs among a number of strains (Fig. 3b; blue boxes). L1_1322p2, L2b_Canada1, and L3_404-LN had indels. Mutations in B_QH111L occurred within the YtgR-binding site for YtgR, an iron-dependent transcription factor that binds to the IGR and regulates operon expression (29), which is conserved among all other strains (Fig. 3b).

**FIG 4:**
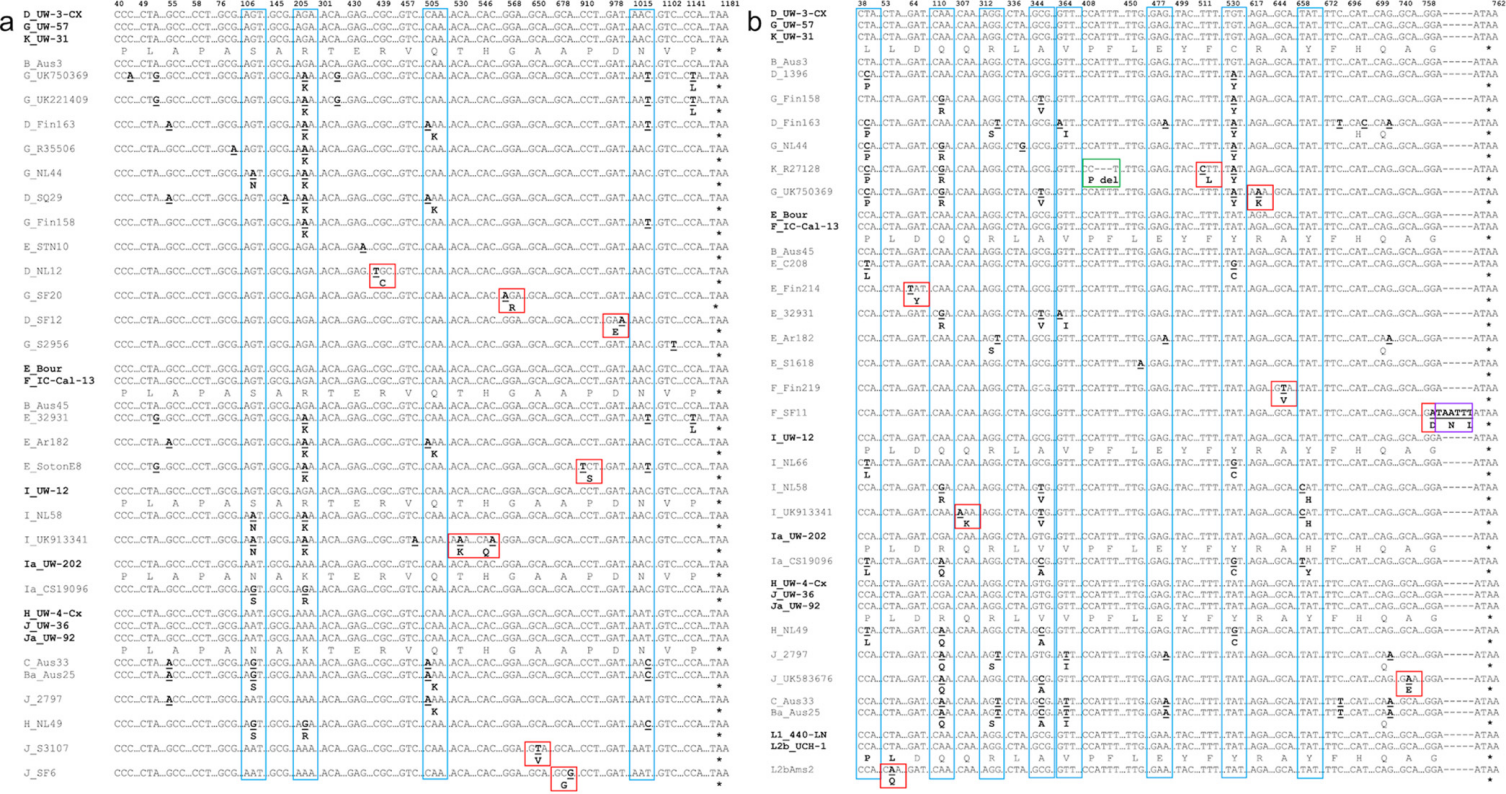
Partial nucleotide sequences of *trp*B (a) and *trp*A (b) urogenital strains with regions of single nucleotide polymorphism (SNPs) and indels in comparison to the 21 *Ct* reference strain sequences (in bold). Australian aboriginal strains Ba_Aus25, B_Aus3, B_Aus45, and C_Aus33 were included with the urogenital strains due to their “true” genome sequence identity as urogenital strains. Homologous regions in the sequence are not shown and are denoted as “….” Dashes denote nucleotide deletion(s). Nucleotides in bold denote substitutions, and bold aa letters denote nonsynonymous aa substitutions compared to the reference strain. Nucleotide and/or aa changes that are unique to a single strain are boxed in red; changes common across several sequences/variants are boxed in blue. “ins,” insertion (boxed in purple); “fs,” frameshift; “ext,” extension; “del,” deletion (boxed in green).

**FIG 5:**
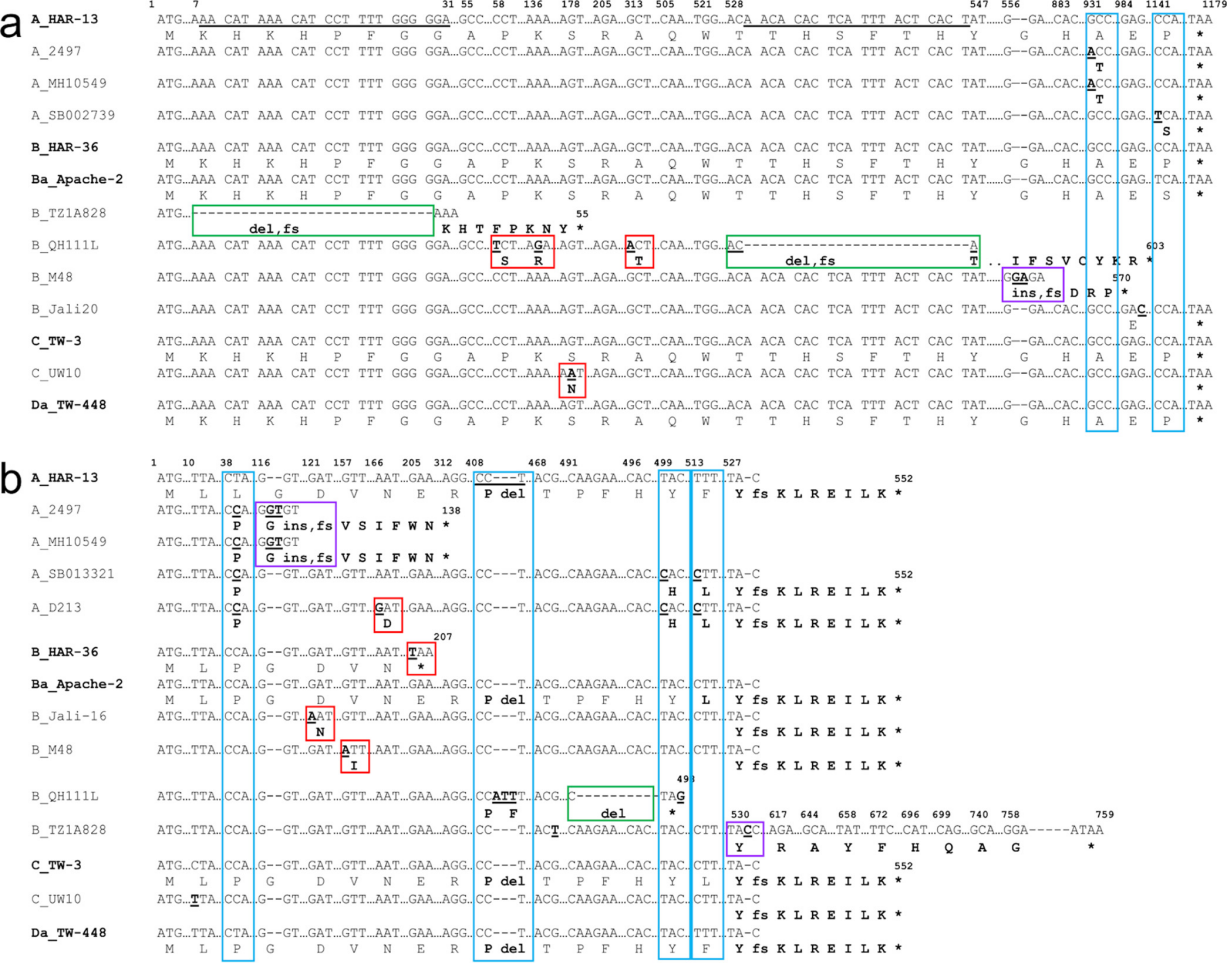
Partial nucleotide sequences of *trp*B (a) and *trp*A (b) ocular strains with regions of single nucleotide polymorphism (SNPs) and indels in comparison to the 21 *Ct* reference strain sequences (in bold). Australian aboriginal strains Ba_Aus25, B_Aus3, B_Aus45, and C_Aus33 were included with the urogenital strains due to their “true” genome sequence identity as urogenital strains. Homologous regions in the sequence are not shown and are denoted as “….” Dashes denote nucleotide deletion(s). Nucleotides in bold denote substitutions, and bold aa letters denote nonsynonymous aa substitutions compared to the reference strain. Nucleotide and/or aa changes that are unique to a single strain are boxed in red; changes common across several sequences/variants are boxed in blue. “ins,” insertion (boxed in purple); “fs,” frameshift; “ext,” extension; “del,” deletion (boxed in green).

In *trp*B, there were five common nt positions with nonsynonymous substitutions across urogenital strains (Fig. 4a; blue boxes) while six urogenital strains had unique nonsynonymous substitutions (Fig. 4a; red boxes) as did three ocular strains (Fig. 5a, green and purple boxes; Table S3). Two nonsynonymous mutations were common across the ocular strains (Fig. 5a, blue boxes; Table S3). The indel in B_QH111L resulted in a frameshift and early truncation at nt position 624. There were no SNPs for LGV strains.

10.1128/mBio.00605-21.9 TABLE S4 Nucleotide polymorphisms in *trp*A for C. trachomatis clinical and reference ocular strains. Download Table S4, PDF file, 0.09 MB. Copyright © 2021 Bommana et al. 2021 Bommana et al.

*trp*A ocular and urogenital strains exhibited the highest overall nt diversity (Table S4). Seven urogenital and four ocular strains had nt positions with nonsynonymous substitutions across a number of strains (Fig. 4b and Fig. 5b, blue boxes; Table S4). Unique nonsynonymous substitutions were noted for 10 of these strains (Fig. 4b and Fig. 5b, red, green, and purple boxes; Table S4). We previously noted that F_SF11 (i.e., F_I-IV) had a nonsynonymous substitution and 2-aa extension exhibiting decreased operon function *in vitro* (26). Most ocular strains had a deletion at nt position 531 with frameshift and early truncation of the protein at 184 aa as previously described (25). Interestingly, 410_412delATT was also commonly observed (Fig. 5b; Table S4). Four other strains also had indels. B_TZ1A828 was the only ocular strain with an intact TrpA-CDS of 762 nt (Fig. 5b; Table S4).

### Ocular and urogenital but not LGV strains are under positive selection.

Details on polymorphic sites, haplotype diversity, and Pi(a)/Pi(s) ratios for TrpR-, TrpB-, and TrpA-CDS are shown in Table 1. TrpA and TrpB ocular strains were under positive selection, as was TrpA for the urogenital strains (Table 1). While both vaginal and urethral TrpA-CDS were under positive selection, the latter exhibited greater nt diversity and a higher Pi(a)/Pi(s) ratio, although both had higher diversity and ratios compared to ocular strains (Table 1). TrpR- and TrpB-CDS appeared to be under negative selection for the urogenital strains. LGV strains had 0 to 1 segregation sites (Ss), and therefore, Pi(a)/Pi(s) values could not be calculated.

**TABLE 1:** Nucleotide diversity and Pi(a)/Pi(s) ratio for C. trachomatis TrpR-, TrpB-, and TrpA-coding sequences (CDS) for ocular, urogenital/rectal, and LGV strains based on phylogenetic lineages^*a*^

CDS	No. of haplotypes	Haplotype diversity (Hd)	*Ct* lineage	No. of sequences	No. of polymorphic segregating sites (Ss)	Synonymous nucleotide diversity (Pi(s); Jukes-Cantor corrected)	Nonsynonymous nucleotide diversity (Pi(a); Jukes-Cantor corrected)	Pi(a)/Pi(s) ratio	No. of synonymous substitutions	No. of nonsynonymous substitutions
*trp*R	10	0.2889	Ocular	79	1	0	0.00011	0	0	1
			Urogenital/rectal	455	14	0.00179	0.00101	0.564	4	9
			LGV	70	0	0	0	0	0	0
*trp*B	26	0.4844	Ocular	79	36	0.00075	0.00164	2.182	16	20
			Urogenital/rectal	455	20	0.00126	0.00092	0.726	9	11
			LGV	70	1	0	0	0	1	0
*trp*A	19	0.5883	Ocular	79	8	0.00074	0.00164	2.212	3	5
			Urogenital/rectal	455	16	0.00158	0.00209	1.317	6	10
			LGV	70	0	0	0	0	0	0

a Note that TrpR-, TrpB-, and TrpA-CDS were extracted from individual strains and segregated into ocular, urogenital, rectal, and LGV disease groups; TrpA-CDSs from urogenital strains were further segregated into vaginal and urethral strains. Diversity metrics were calculated in DnaSP6 using the MAFFT alignment (see Materials and Methods).

### TrpR mutant strains reveal structural and functional changes in the DNA-binding motif and ligand-binding sites of the symmetric dimer.

*Ct* TrpR forms a symmetric dimer of two chains each consisting of 94 aa and four identical ligand-binding sites (LBSs). Each subunit contains six α-helices (i.e., A to F; D and E; and DE turn, termed DNA-binding motif [DBS]) that undergo conformational change upon l-tryptophan binding to the LBSs, which is necessary for binding to the operator DNA (30). Three biologically fundamental interactions, protein-protein (dimerization), protein-ligand (corepressor binding), and protein-DNA (operator binding), are noted in Fig. S2.

10.1128/mBio.00605-21.2 FIG S2 Crystal structure of tryptophan repressor in E. coli, PDB: 6ENI. The two monomer subunits of the symmetric dimer are shown as cartoon diagrams, chain A in cyan and chain B in pink. Two molecules of indole-3-acetic acid (IAC) is bound at chain A and chain B; the 1,2-ethanediol (EDO) is bound at chain A only. The DNA-binding motif (DBS) involved in the protein-DNA (operator binding) interactions is shown in orange and is present in chains A and B. The putative ligand-binding sites for IAC and EDO are shown in blue. The reference sequence of E. coli TrpR 6eni with the LBS (blue), DBS (orange), and residues involved in dimerization (bold) is shown as well. Each molecule of the ligand IAC has LBS on chain A and chain B, respectively. Download FIG S2, PDF file, 0.1 MB. Copyright © 2021 Bommana et al. 2021 Bommana et al.

**FIG 6:**
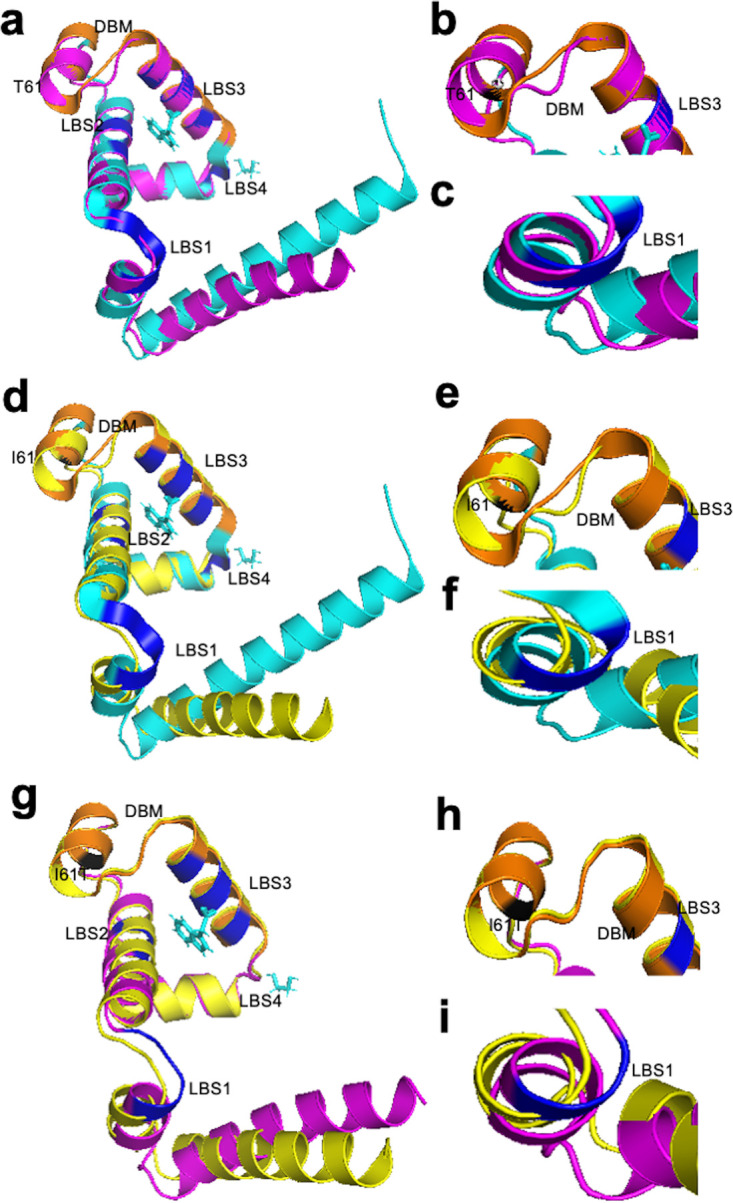
TrpR 3D predicted structures of *Ct* clinical E_R27091 and reference E_Bour strains (a to i). (a) TrpR structure of mutant strain E_R27091 (magenta) superimposed on the template 6eniA (cyan), with DNA-binding motif (DBM) in orange. The putative ligand-binding sites (LBS) 1, 2, and 3 are shown in dark blue, and the aa substitution in relation to E_Bour at T61 is in black. (b and c) Structural changes in DBM and LBS1, respectively, of the mutant are shown. (d) TrpR structure of E_Bour (yellow) superimposed on the template 6eniA (cyan) with annotations as per panels a, b, and c. (e and f) Structural changes in DBM and LBS1 of E_Bour. (g to i) TrpR 3D predicted structures of E_R27091 superimposed on E_Bour (g) with structural changes noted in relation to DBM based on I61T aa substitution (h) and E_Bour at LBS1 (i).

**FIG 7:**
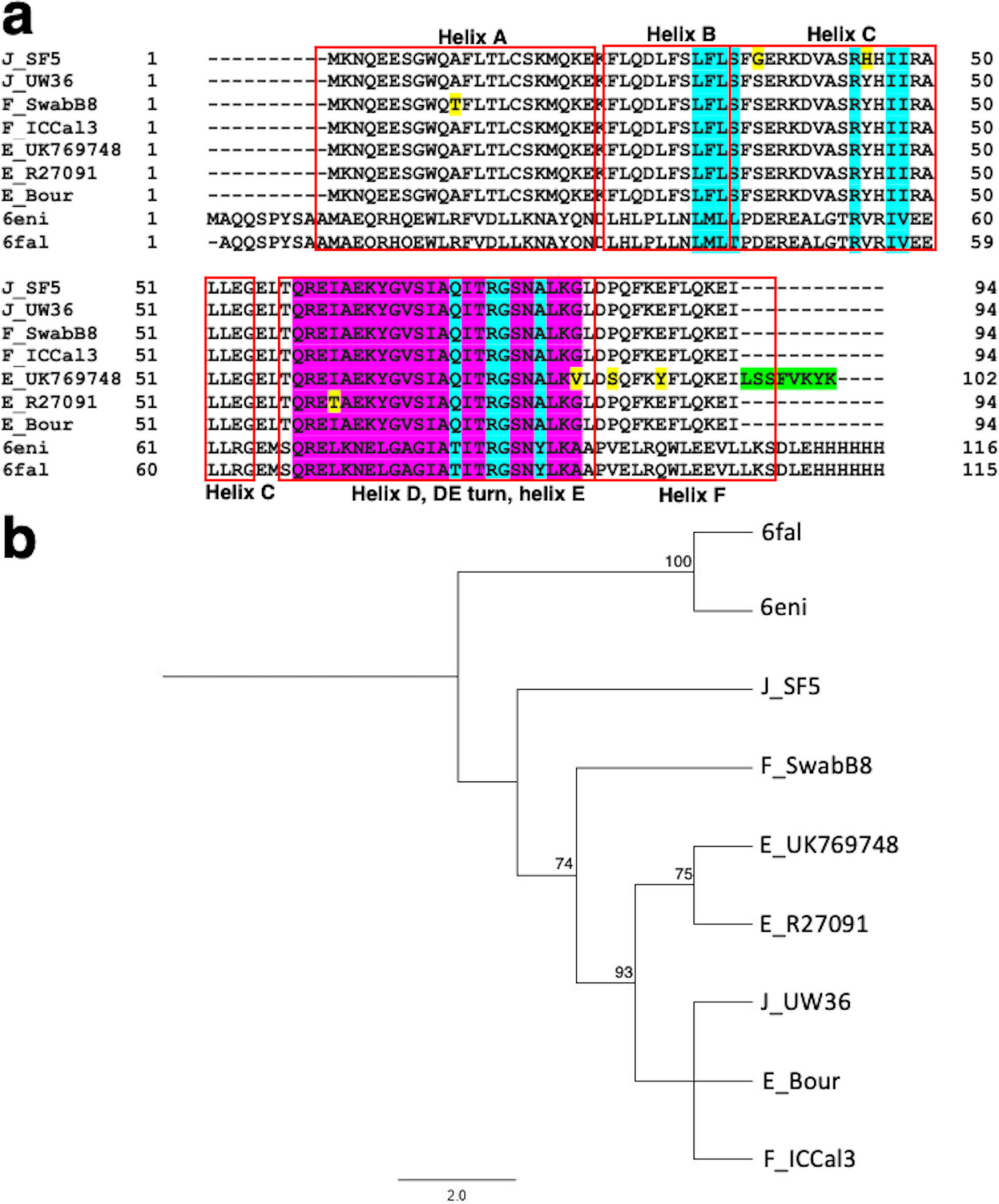
TrpR amino acid alignment (a) and phylogeny (b) of *Ct* mutant strains (E_R27091, E_UK769748, F_SwabB8, and J_SF5), reference strains (E_Bour, F_IC-Cal-3, and J_UW-36) and Protein Data Bank (PDB) templates (6fal and 6eni). (a) TrpR aa alignments were created using Clustal Omega, and the residues involved in putative ligand-binding sites (cyan), DNA-binding motif (magenta), and helices (box outlined in red) were annotated based on the crystal structures of Escherichia coli 6fal and 6eniA available from PDB and NCBI. Amino acid substitutions (yellow) and extension (green) in the mutant strains relative to their reference strain are annotated. (b) TrpR phylogeny of *Ct* strains and templates; the PDB aa templates were constructed using the approximate maximum likelihood phylogenetic tree in FastTree 2.1.11 with a generalized time-reversible model with 1,000-bootstrap sampling. Scale bar represents the number of differences or distance between the sequences.

Since there are no crystal structures of *Ct* TrpR, the protein was modeled against the best-hit homology to known crystal structure templates. Three TrpR urogenital strain mutants (i.e., E_R27091, F_SwabB8, and J_SF5) and their respective reference strains (i.e., E_Bour, F_ICCal3, and J_UW36 [Fig. 6 and Fig. S3 and S4; magenta]) had >95% correct folds and a GA341 MODELLER score of 1.0 to Escherichia coli variant T44L S88Y 6eni, indicating a highly reliable model (31, 32). Indole acetic acid (IAC) was found to be the bound ligand. The highest identity to these strains was 33% with an overall root mean square deviation (RMSD) of 2.5 Å for mutant and 4.065 Å for reference strains. E_UK769748 had a GA341 MODELLER score of 0.99 with highest homology to E. coli 6fal TrpR with an identity of 33%. TrpR *Ct* strain alignments with the templates 6eni and 6fal are shown in Fig. 7a along with the LBSs and DBS residues. Phylogenetic reconstruction of *Ct* strains and the templates is shown in Fig. 7b.

10.1128/mBio.00605-21.3 FIG S3 TrpR 3D predicted structures of *Ct* clinical F_SwabB8 and reference F_IC-Cal-3 strains (A to I). (A) TrpR structure of *Ct* mutant strain F_SwabB8 (magenta) superimposed on the template 6eniA (cyan), with DBM in orange. The LBSs 1, 2, 3, and 4 are shown in dark blue, and the aa substitution in relation to F_IC-Cal-3 at T11 is in black. (B and C) Structural changes in DBM and LBS1, respectively, of the mutant are shown. (D) TrpR structure of F_IC-Cal-3 (yellow) superimposed on the template 6eniA (cyan) with annotations as per panels A, B, and C. (E and F) Structural changes in DBM and LBS1 of E_Bour. (G to I) TrpR 3D predicted structures of F_SwabB8 superimposed on F_IC-Cal-3 (G) with structural changes noted in relation to DBM based (H) and LBS1 (I). Download FIG S3, PDF file, 0.2 MB. Copyright © 2021 Bommana et al. 2021 Bommana et al.

10.1128/mBio.00605-21.4 FIG S4 TrpR 3D predicted structures of *Ct* clinical J_SF5 and reference J_UW-36 strains (A to I). (A) TrpR structure of *Ct* mutant strain J_SF5 (magenta) superimposed on the template 6eniA (cyan), with DBM in orange. The LBSs 1, 2, 3 and 4 are shown in dark blue, and the aa substitution in relation to J_UW-36 at G36 and H45 is in black. (B and C) Structural changes in DBM and LBS1, respectively, of the mutant are shown. (D) TrpR structure of J_UW-36 (yellow) superimposed on the template 6eniA (cyan) with annotations as per panels A, B, and C. (E and F) Structural changes in DBM and LBS1 of J_UW-36. (G to I) TrpR 3D predicted structures of J_SF5 superimposed on J_UW-36 (G) with structural changes noted in relation to J_UW-36 at LBS1 and DBM based on S36G and Y45H aa substitution (H and I). Download FIG S4, PDF file, 0.2 MB. Copyright © 2021 Bommana et al. 2021 Bommana et al.

E_R27091, J_SF5, F_SwabB8, and reference strains with template models are shown in Fig. 5 and Fig. S3 and S4. The E_R27091 I61T mutation was located in the DBM (Fig. 5a and b; charcoal gray), F_SwabB8 A11T was in helix A (Fig. S3; charcoal gray, Fig. 7a), and J_SF5 Y45H was proximal to LBS2 (Fig. S4; charcoal gray, Fig. 7a), while S36G (Fig. S4; charcoal gray) was located further upstream. E_UK769748 could not be modeled due to the aa elongation.

The effect of mutations on protein stability, interactions with single-stranded DNA (ssDNA) (operator-DNA), and protein-ligand affinity was predicted using mCSM and mCSM-lig, respectively (Table 2). There was a predicted decrease in affinity of LBS for IAC, except for E_R27091, but an increased affinity for ssDNA, except for J_SF5 S36G, which would affect its ability to prevent transcription initiation of *trp*BA. These mutations are crucial in affecting repressor activity by altering its affinity with either the operator-DNA or the ligand IAC. There was also a predicted decrease in affinity of LBS for EDO (ethanediol), except E_R27091, suggesting that this mutation is neutral in terms of functional impact. The majority of the mutations were, therefore, predicted to decrease the overall stability of TrpR mutant proteins (Table 2).

**TABLE 2:** Predicted effects of urogenital C. trachomatis TrpR, TrpB, and TrpA mutant strains on protein stability, protein interaction with ssDNA, and ligand affinity noted as change in Gibbs free energy (ΔΔ*G*) and log (affinity fold change)^*d*^

Mutant	Mutation	Mutation location	Effect of mutation on protein stability (ΔΔ*G*)	Effect of mutation on protein interaction with ssDNA (ΔΔ*G*)	Effect of mutation on protein-ligand affinity (log[affinity fold change])	
TrpR^*a*^					EDO	IAC
E_UK769748	G81V	DNA-binding motif (DBM)	0.72	0.49	−1.83	−1.083
	P84S	Ligand-binding site (LBS)	0.49	1.096		−0.153
	E88Y	Helix F	−0.74	0.434	−2.38	−2.207
E_R27091	I61T	DNA-binding motif (DBM)	−3.19	1.078	0.36	0.345
F_SwabB8	A11T	Helix A	−2.05	1.566	−0.83	−0.682
J_SF5	S36G	Helix C	0.08	−1.538	−0.7	−0.239
	Y45H	Helix C	−0.34	0.38	−1.41	−1.404
TrpB^*b*^					PLP	Na^+^
D_NL12	R147C	β-COMM domain	−0.885		−0.49	−1.19
D_SF12	D326E	β-subunit	−0.705		0.11	−1.016
E_SotonE8	R69K	β-subunit	−1.01		−0.47	−1.702
	P304S	β-subunit	−2.228		0.75	−0.339
	P381L	β-COMM domain	−0.677		0.19	−0.773
G_SF20	G190R	β-subunit	−0.634		−0.49	−0.798
I_UK913341	S36N	β-subunit	−0.239		0.39	−0.303
	R69K	β-subunit	−1.01		−0.47	−1.702
	T177K	Proximal to β-subunit interface residues	−0.771		0.5	−0.493
	H182Q	β-COMM domain	−1.852		−0.06	−0.176
J_SF5	A19T	TrpA interaction	−1.105		0.78	−0.285
	N36S	β-subunit	−0.41		−0.61	−1.307
J_S3107	N36S	β-subunit	−0.41		−0.61	−1.307
	A217V	β-subunit	−0.198		0.23	-0.438
TrpA^*c*^					MLI	FMT
E_Fin214	D22Y	α-subunit	−0.384		−0.56	−0.725
F_Fin219	A215V	α-subunit	−0.489		−0.39	−0.488
F_SF11	G253D	α-subunit	−0.478		2.05	1.481
G_UK750369	L13P	α-subunit	−1.334		−0.18	−0.442
	Q37R	α-subunit	0.147		−0.3	−0.447
	A115V	α-subunit	−0.435		−0.3	−0.436
	C177Y	α-L6	−0.473		−0.21	−0.366
	R206K	Catalytic domain	−1.396		−0.88	−0.88
I_UK913341	Q37R	α-subunit	0.227		−0.3	−0.454
	Q103K	α-subunit	−0.172		−0.29	−0.62
	A115V	α-subunit	−0.386		−0.3	−0.436
	E178Q	α-L6	−0.757		−0.724	−0.723
	Y220H	α-subunit	−1.576		−0.35	−0.532
J_UK583676	A247E	α-subunit	−0.606		0.71	0.454

a See Fig. 5 for location and position of the mutation in the TrpR amino acid sequence.

b See Fig. 6 for location and position of the mutation in the TrpB amino acid sequence.

c See Fig. 9 for location and position of the mutation in the TrpA amino acid sequence.

d Abbreviations: EDO, ethanediol; IAC, indole acetic acid; PLP, pyridoxal 5′-phosphate; Na^+^, sodium ion; MLI, malonate ion; FMT, formic acid.

### TrpB and TrpA mutant strains reveal structural and functional changes in protein-ligand affinity affecting tryptophan synthase function.

The αββα tetramer is responsible for two steps in tryptophan biosynthesis. The α-subunit catalyzes aldolytic cleavage of indole-3-glycerol phosphate (IGP) to glyceraldehyde 3-phosphate (GAP) and indole; indole is transferred via an intermolecular 25-Å-long hydrophobic tunnel to the active site of the β-subunit where it condenses with l-serine in a pyridoxal phosphate (PLP)-dependent reaction to produce l-tryptophan and water (24). The reactions occur when α- and β-subunits are in the closed conformation to mutually activate each other. Formation of the indole tunnel involves residues α176 to α196 that trap indole and is dependent on TrpA α-Loop 6 (α-L6) mobility for allosteric interaction with TrpB beta communication (β-COMM) domain (33).

**FIG 8:**
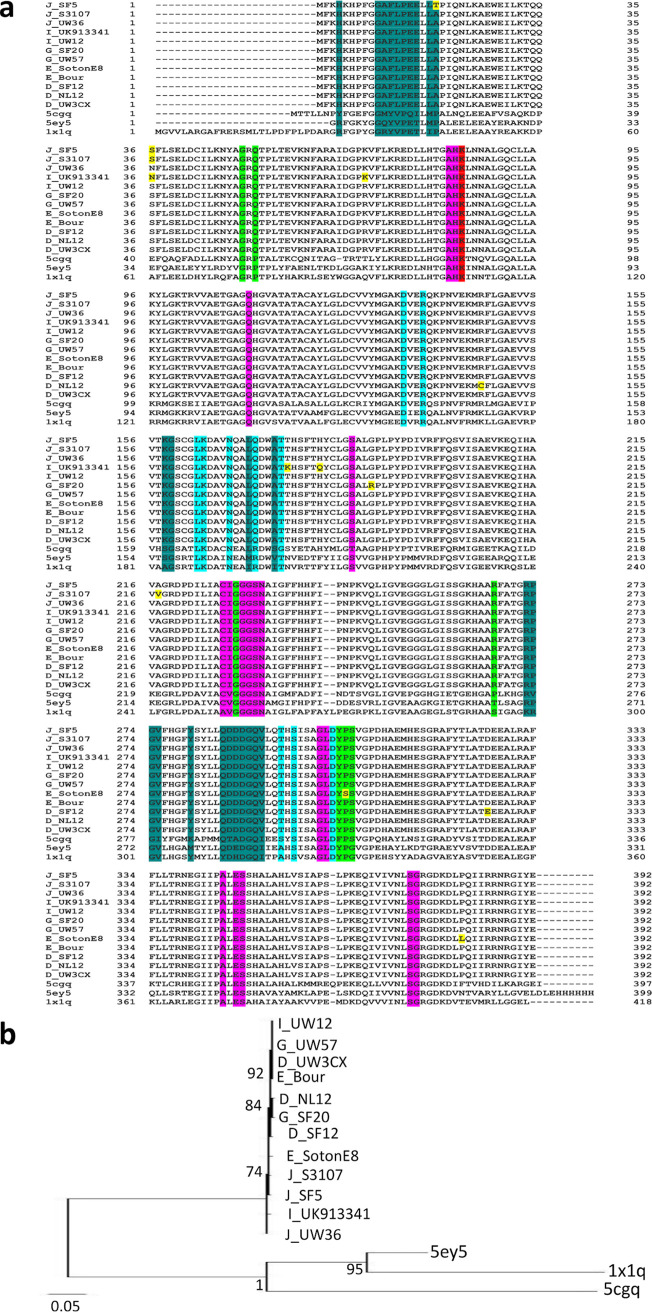
TrpB amino acid alignment (a) and phylogeny (b) of *Ct* mutant strains (D_NL12, D_SF12, E_SotonE8, G_SF20, I_UK913341, J_S3107, and J_SF5), reference strains (D_UW3CX, E_Bour, G_UW57, I_UW12, and J_UW36) and Protein Data Bank (PDB) templates (5cgq, 5ey5, and 1x1q). (a) TrpB aa alignments were created using Clustal Omega, and the residues involved in Na^+^ ion and pyridoxal 5′-phosphate interaction (green and magenta), catalytic activity (red), TrpA interaction (dark green), and interface of α-Loop 6 (α-L6) and monovalent cation (MVC)-binding loop (cyan) were annotated based on the crystal structures and sequence of 5ey5 from PDB and NCBI. Amino acid substitutions (yellow) in the mutant strains relative to their reference strain are also annotated. (b) TrpA phylogeny of *Ct* strains and templates was constructed using the approximate maximum likelihood phylogenetic tree in FastTree 2.1.11 with a generalized time-reversible model with 1,000-bootstrap sampling.

**FIG 9:**
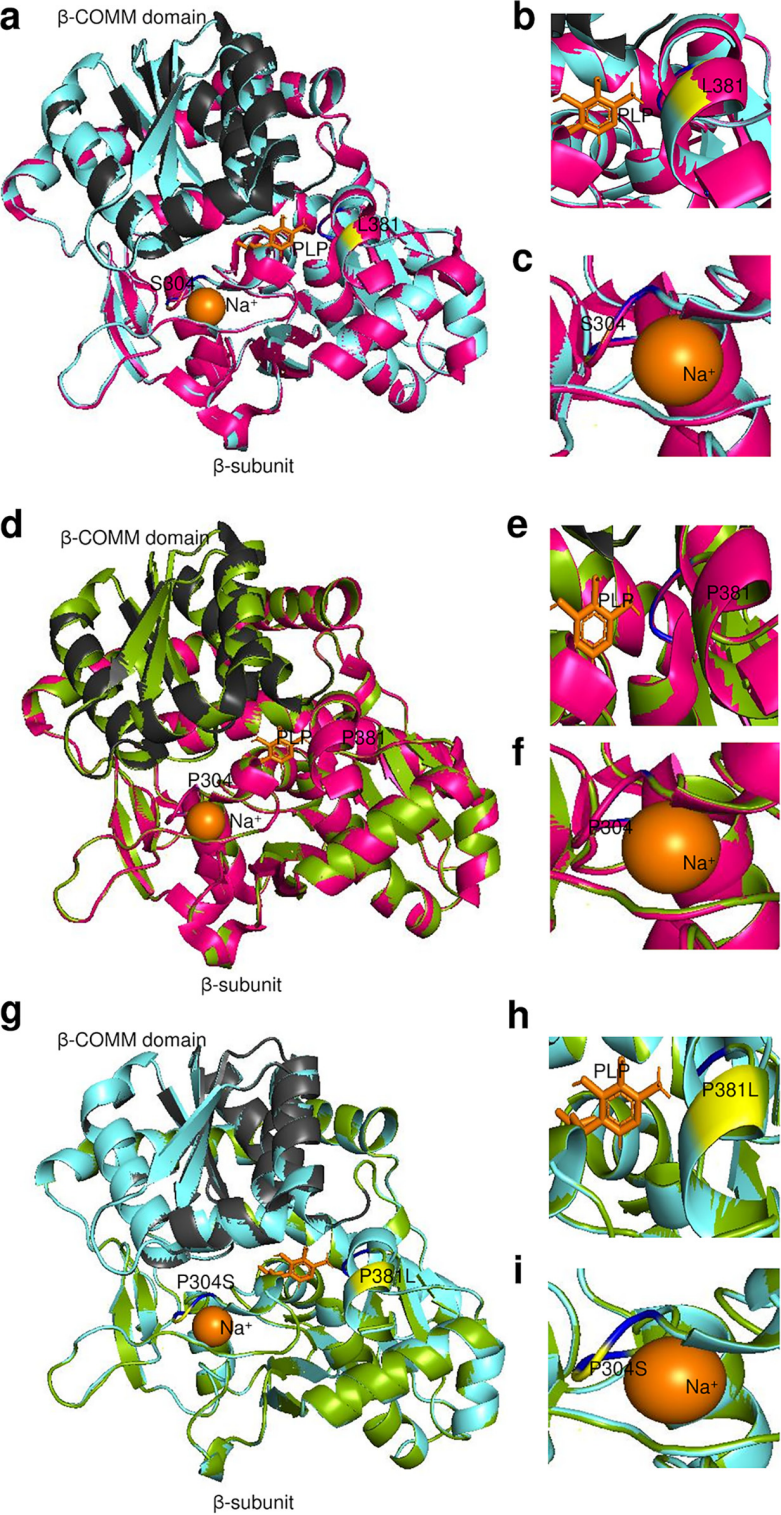
TrpB 3D predicted structures of *Ct* clinical E_SotonE8 and reference E_Bour strains (a to i). (a) TrpB structure of mutant strain E_SotonE8 (cyan) superimposed on the template 5ey5 (magenta), with the β-COMM domain in charcoal gray. Na^+^ and the cofactor pyridoxal phosphate (PLP) interacting residues are shown in blue, and the aa substitution in relation to E_Bour at S304 and L381 is in yellow. (b and c) Structural changes in Na^+^ and the cofactor PLP interacting residues of the mutant E_SotonE8 are shown. (d) TrpB structure of E_Bour (green) superimposed on the template 5ey5 (magenta) with annotations as per panels a, b, and c. (e and f) Structural changes in Na^+^ and the cofactor PLP interacting residues of E_Bour. (g to i) TrpB predicted structures of E_SotonE8 superimposed om E_Bour (g) with structural changes noted in relation to PLP interacting residue on P381L (h) and Na^+^ interacting residue on P304S (i).

For TrpB, seven urogenital mutant strains and their reference strains had the highest homology with the last bacterial common ancestor (LBCA), 5ey5, with >95% correct folds and a GA341 MODELLER score of 1.0 with 57% identity to *Ct* strains and an overall RMSD of <1 Å; alignments including templates 5ey5, 5cgq, and 1x1q are shown in Fig. 8a. Sodium ion (Na^+^) and PLP were identified as the bound ligands for the monovalent cation (MVC)-binding site. Phylogenetic reconstruction of *Ct* strains and the templates is shown in Fig. 8b. Models are shown for E_SotonE8 βP304S (MVC-binding site), I_UK913341 βT177K, and βH182Q (β-COMM domain with βT177K proximal to residues interacting with α-subunit) in Fig. 9a to c and Fig. S5A to C but not for D_NL12 βR147C in the β-COMM domain or mutations in D_SF12, G_SF20, J_S3107, and J_SF5 due to space limitations. All mutations predicted a decrease in protein-ligand affinity for Na^+^, while seven mutations showed reduced affinity for PLP but resulted in reduced protein stability for all mutant strains (Table 2).

10.1128/mBio.00605-21.5 FIG S5 TrpB 3D predicted structures of *Ct* clinical I_UK913341 and reference I_UW12 strains (A to I). (A) TrpB structure of mutant strain I_UK913341 (cyan) superimposed on the template 5ey5 (magenta), with the COMM domain in charcoal gray. TrpB interface residues that interact with TrpA are shown in blue, and the aa substitution in relation to E_Bour at K177 and Q182 is in yellow. (B and C) Structural changes in β-subunit interface and COMM domain residues of the mutant E_SotonE8 are shown. (D) TrpB structure of I_UW12 (green) superimposed on the template 5ey5 (magenta) with annotations as per panels A, B, and C. (E and F) Structural changes in β-subunit interface and COMM domain residues of the reference I_UW12 are shown. (G to I) TrpB predicted structures of I_UK913341 superimposed on I_UW12 (G) with structural changes noted in relation to β-subunit interface residues on P381L (H) and COMM domain on P304 (I). Download FIG S5, PDF file, 0.3 MB. Copyright © 2021 Bommana et al. 2021 Bommana et al.

Analyses performed in Arpeggio revealed that wild-type residue P304 forms a hydrogen bond with L284 and Y304 and repulsive van der Waals clash with Na^+^ to activate the β-subunit (see Fig. 12a). The E_SotonE8 P304S results in loss of van der Waals clash with Na^+^ and a weaker affinity of the β-subunit for Na^+^ in addition to loss of hydrogen bonding with Y303 (see Fig. 12b), suggesting loss of β-subunit activation. Wild-type residue T177 is proximal to residues interacting allosterically with the α-subunit. T177 makes a polar van der Waals clash with S179, F180, and D173, weak polar van der Waals clash with T181 and D173, and a van der Waals clash with S179 and R102, in addition to a carbonyl and proximal bond with W174 (see Fig. 12c). W174 and D173 are β-subunit residues involved in allosteric interactions with the α-subunit (see Fig. 12c, cyan). The I_UK913341 T177K results in loss of interaction with R102 and all polar interactions with D173, S179, and T181 (see Fig. 12d), indicating suboptimal allosteric interactions with the α-subunit.

**FIG 10:**
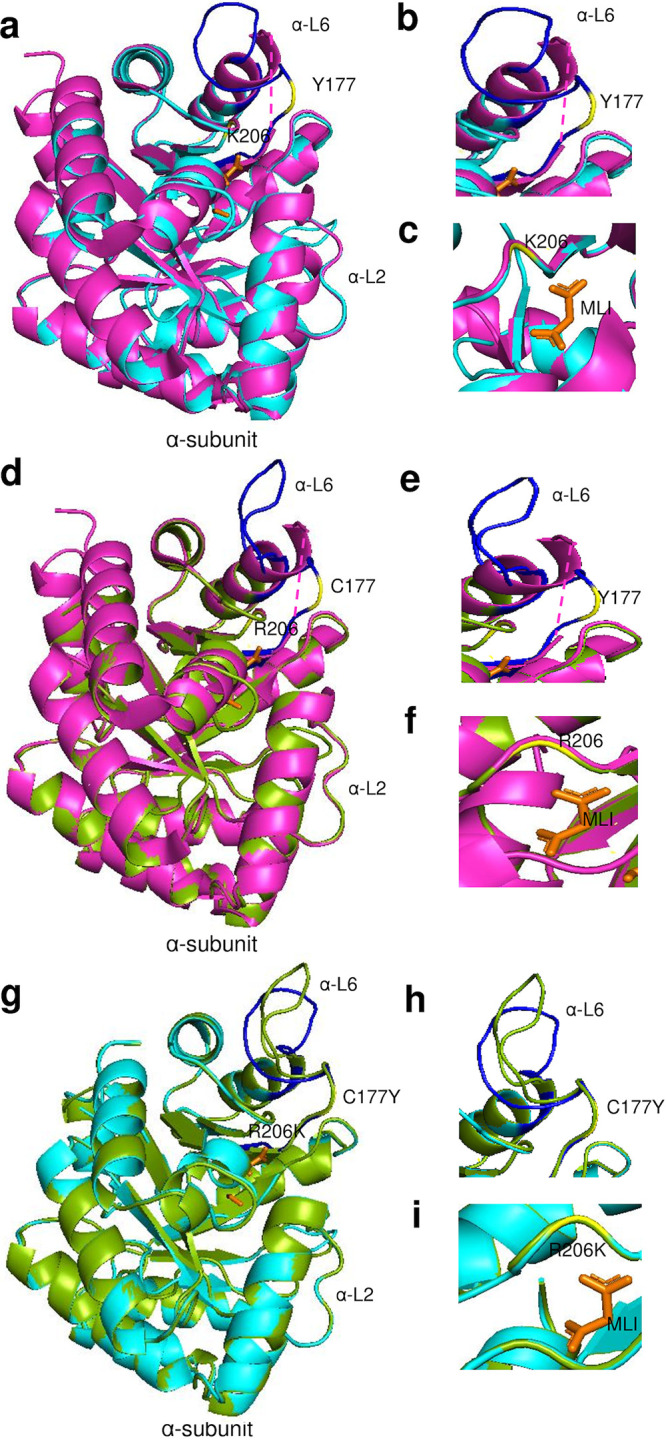
TrpA 3D predicted structures of *Ct* clinical G_UK750369 and reference G_UW57 strains (a to i). (a) TrpA structure of mutant strain G_UK750369 (cyan) superimposed on the template 5tch (magenta), with α-Loop 6 (α-L6) of the α-subunit in blue and malonate ion (MLI) in orange. The aa substitution in relation to G_UW57 at Y177 and K206 is in yellow. (b and c) Structural changes and location of the α-L6 and catalytic and subunit interaction regions of the mutant G_UK750369 are shown. (d) TrpA structure of G_UW57 (green) superimposed on the template 5tch (magenta) with annotations as per panels a, b, and c. (e and f) Structural changes in the α-L6 and catalytic and subunit interaction regions of the reference G_UW57. (g) TrpA predicted structures of G_UK750369 superimposed on G_UW57 (g) with structural changes noted in relation to α-L6 based on C177Y aa substitution (h) and catalytic and subunit interaction region based on R206K (i).

**FIG 11:**
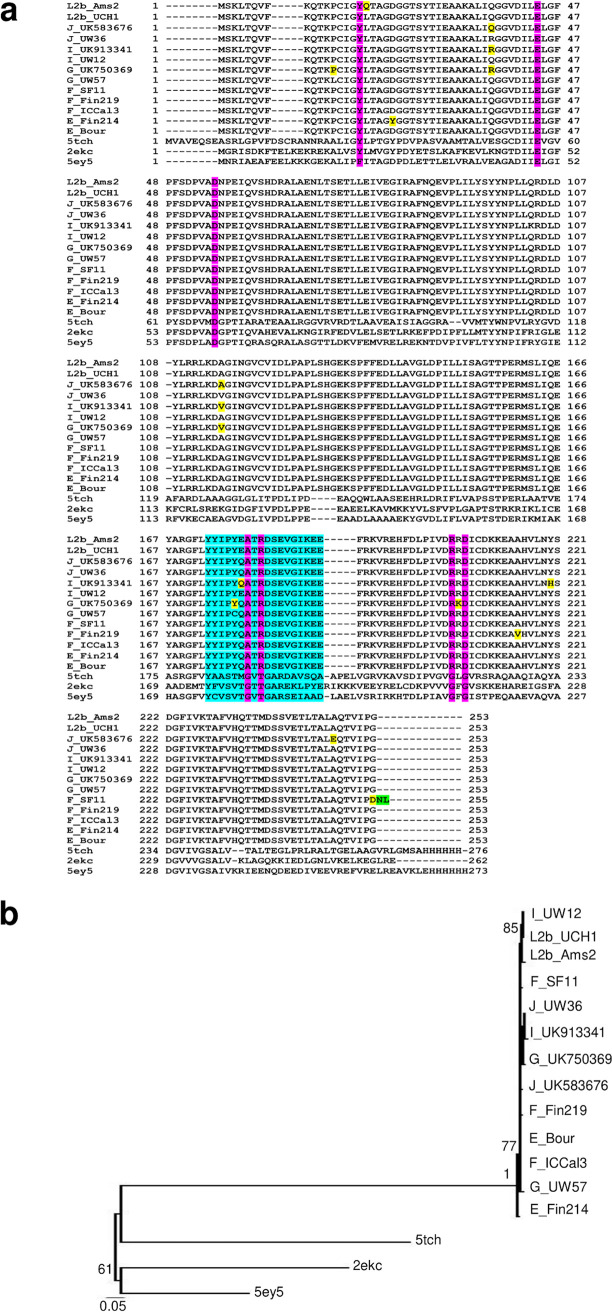
TrpA amino acid alignment (a) and phylogeny (b) of *Ct* mutant strains (E_Fin214, F_Fin219, F_SF11, G_UK750369, I_UK913341, J_UK583676, and L2b_Ams2), reference strains (E_Bour, F_ICCal3, G_UW57, I_UW12, J_UW36, and L2b_UCH1), and Protein Data Bank (PDB) templates (5tch, 5ey5, and 2ekc). (a) TrpA aa alignments were created using Clustal Omega, and the residues involved in catalytic and subunit interaction (magenta), α-L6 residues α176 to 196, were annotated based on the crystal structures and sequence of 5tch from PDB and NCBI. Amino acid substitutions (yellow) and extension (green) in the mutant strains relative to their reference strain are also annotated. (b) TrpA phylogeny of *Ct* strains and templates was constructed using the approximate maximum likelihood phylogenetic tree in FastTree 2.1.11 with a generalized time-reversible model with 1,000-bootstrap sampling.

TrpA protein structures were modeled for seven urogenital strains and their respective reference strains, and all, except for G_UK750369 and G_UW57, had the closest homology to Aquifex aeolicus VF5 2ekc with >95% correct folds and a GA341 MODELLER score of 1.0, which had 32% identity to *Ct* strains; alignments with templates 5tch, 5ey5, and 2ekc are shown in Fig. 11a below. G_UK750369 and G_UW57 had the closest homology to Mycobacterium tuberculosis 5tch with a GA341 MODELLER score of 1.0 and sequence homology of 32% with an overall RMSD of 2.6 Å (Fig. 10a and d, magenta). Malonate ion (MLI) and formic acid (FMT) were identified as the bound ligands for the catalytic subunit. The phylogenetic tree is shown in Fig. 11b.

Modeling G_UK750369 αC177Y and αR206K located in *α*-L6 (Fig. 11a; also Fig. 10a and b, yellow) that are catalytic and binding sites for MLI (Fig. 11a; also Fig. 10a and c, yellow) resulted in structural changes in α-L6 and the catalytic site compared to G_UW57 (Fig. 10g to i) and 5tch (Fig. 10d, e, and f). I_UK913341 αE178Q located in α-L6 and mutations in E_Fin214, F_Fin219, F_SF11, and J_UK583676 were located elsewhere on the protein (Fig. 11a). All mutations resulted in reduced affinity for ligands MLI and FMT in addition to decreased protein stability, except for αQ37R, which had an increased/neutral effect on protein stability, and αG253D, which had increased affinity for MLI and FMT but reduced protein stability (Table 2).

**FIG 12:**
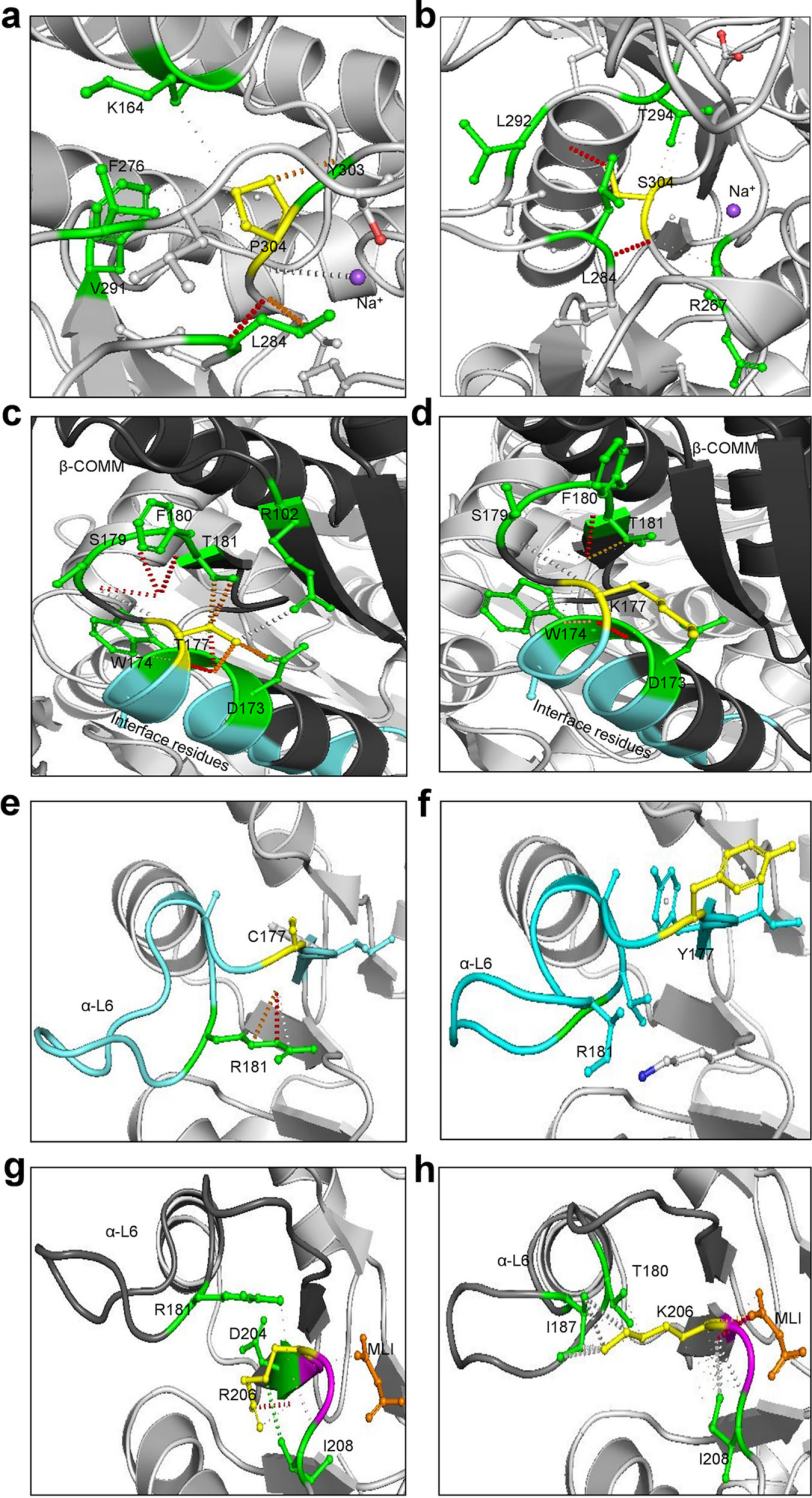
Effect of wild-type and mutant residues on interatomic interactions in TrpA and TrpB protein structures and small-molecule ligand binding. (a and b) The wild-type residue P304 (yellow) of E_Bour β-subunit (a) and the mutant residue S304 (yellow) of E_SotonE8 β-subunit (b) interactions with neighboring residues (green) and the ligand Na^+^ (purple). (c and d) The wild-type residue T177 (yellow) of I_UW12 β-subunit (c) and the mutant residue K177 (yellow) of I_UK913341 β-subunit (d) interacting with αβ interface (cyan) and neighboring residues (green). (e and f) The wild-type residue C177 (yellow) of G_UW57 α-subunit (e) and mutant residue Y177 (yellow) of G_UK750369 α-subunit (f) interactions with residue R181 (green) located in the α-L6 (cyan). (g and h) The wild-type residue R206 (yellow) of G_UW57 α-subunit (g) and mutant residue K206 (yellow) of G_UK750369 α-subunit (h) interactions with residues (green) in the α-L6 and malonate ion (MLI; orange). The α-L6 region shown in charcoal gray and catalytic site in magenta. Polar contacts (red), weak polar contacts (orange), van der Waals clash interactions (gray), and hydrophobic van der Waals contact (green) between residues and ligands are shown as dotted lines.

Wild-type residue C177 forms polar contacts, weak polar contacts, and van der Waals clash interactions with the side chain of R181 (Fig. 12e). G_UK750369 C177Y resulted in loss of all interactions with R181 and any surrounding residues (Fig. 12f) with reduced affinity to MLI and FMT and decreased protein stability (Table 2). Interestingly, the Y177 residue was present in all *Ct* reference and mutant strains (Fig. 11a), suggesting that urogenital *Ct* strains inherently have compromised TS function.

Wild-type residue R206 located in the catalytic and binding site for MLI as per 5tch forms polar contacts (Fig. 12g; orange dotted lines) with MLI, hydrophobic van der Waals contact with the side chain of I208, polar van der Waals clash with D204, and polar contacts with the side chain of R181 located in α-L6 (Fig. 12g). R206K forms polar bonds and gained van der Waals clashes with MLI, lost the polar contact with R181 in α-L6 and D204, and gained van der Waals clashes and weak hydrogen bonds with I187 and T180. It also had weak polar and undefined van der Waals clashes with I208 (Fig. 12h). R206K resulted in reduced affinity to MLI and FMT and decreased protein stability (Table 2).

## DISCUSSION

Human- and animal-pathogenic species of the Chlamydiaceae family such as *Ct*, Chlamydia psittaci, and Chlamydia abortus represent the most evolutionarily reduced genomes compared to family members of *Simkaniaceae*, *Parachlamydiaceae*, and *Waddliaceae* (34). Chlamydiaceae are the most recently evolved member with the smallest chromosome, likely reflecting recent adaptation to a relatively stable, homeostatic environment within mammalian and avian host cells. Other family members have a much wider range of eukaryotic hosts, including protozoans such as amoebae (34). Chlamydiae genomes contain a “plasticity zone” (PZ) (35), which has undergone increased levels of genetic variation compared to other chromosomal regions and includes virulence factors (36) such as the tryptophan operon. Simkania negevensis is the only species that has a complete operon comprised of *trp*R, *trp*Aa-Ab, *trp*B, *trp*D, *trp*C, *trp*Eb-Ea, and *aro*A that likely represents the ancestral operon for the phylum (37).

Comparative genomics of *Ct* strains have shown that ocular strains have emerged at a later time point in evolution than the nonprevalent urogenital strains that appeared ∼10 to 15 million years ago (11). It is thought that, through reductive evolution, ocular strains lost their ability to synthesize tryptophan to maximize fitness and survive in a presumably indole-deficient ocular environment (26, 38). However, more recent ocular microbiome data suggest that indole-producing bacteria such as Propionibacterium acnes and Escherichia coli are commonly present on the ocular surface of the healthy conjunctiva (39, 40). Other mechanisms may therefore be at play.

Analysis of the expanded number of *Ct* strains in the present study revealed an increased diversification of the operon for the less studied *trp*R region and IGR as well as *trp*B and *trp*A. Mutations occurred in archival samples from the early 1900s to early 2000s for ocular samples while most of the mutations in urogenital strains were in the 2000s, spanning 59 years. Our sample set was biased toward prevalent genotypes E, F, and G with some rarer genotypes present as singletons or in smaller numbers (e.g., Ba, C, Da, Ja, L2c, L3). Increased sampling would likely reveal a greater diversity of genomes and delineate timeline-based mutation accumulation in ocular, urogenital, and rectal strains.

We found that the majority of genetic variation consisted of SNPs and indels with no evidence of recombination occurring within the operon, consistent with the relative congruence of the phylogenetic trees. The findings concur with our previous *Ct* genome analyses where, despite evidence for recombination occurring frequently at multiple sites throughout the chromosome (10, 11), it occurred less frequently than mutation (mean ρ/θ of 0.12; 95% credibility interval of 0.10 to 0.23) (16). Although it should be noted that the operon is part of a much larger recombinant fragment (18), our current data indicate that mutation is a significant driving force in the evolution of the operon.

Operon and *trp*A phylogenies were not entirely congruent with the well-described *Ct* whole-genome phylogeny (11, 16), except for the ocular strains. The operon phylogeny revealed a new clade consisting of an admixture of prevalent and nonprevalent urogenital strains with the prevalent clade as the earliest ancestor. Each clade appeared to be undergoing diversification and coevolution with polyphyletic branching of the nonprevalent clade. However, subclades arising from mixed and prevalent urogenital clades were clonal. The ocular strains exhibited a more rapid monophyletic diversification, including, for example, clonal A strains from a single geographic site in Tanzania (16).

Operon SNP analysis revealed six urogenital strains with unique mutations in *trp*R. Strain E_UK769748 had five SNPs with an eight-residue extension at the C terminus of TrpR. These strains were isolated between 2003 and 2012, suggesting recent mutations, although no samples with complete operon sequences were available from these regions prior to 2003. Interestingly, the ocular and LGV TrpR-CDS had relatively high synteny.

B_QH111L had two nonsynonymous aa changes in the IGR at the exact binding site for YtgR, the only known iron-dependent transcription factor in *Chlamydia* (29). YtgR binds to the novel IRG promoter and regulates *trp*B and *trp*A expression during iron starvation. Simultaneously, YtgR binding promotes termination of transcripts from the primary promoter upstream of TrpR29. Although the B_QH111L *trp*BA mutations and resulting protein truncations suggest reductive evolution resulting in loss of TS function, the IGR mutations indicate selective pressure that disables the response to fluctuating iron levels with further loss of operon function.

Eight urogenital strains had novel mutations in *trp*B as did some ocular strains (Fig. 4). Gambian strain B_M48 had an indel with a frameshift and early truncation of the protein. Previously described B_QH111L from China and B_TZ1A828 from Tanzania both had frameshifts and early truncation of the protein (25, 28, 41). These strains were isolated between 1998 and 2016, similar to the time frame for the *trp*R mutations.

*trp*A SNP analyses revealed mutations and indels with frameshifts and early truncation of the CDS similar to previous studies (25, 42) but also novel SNPs for both ocular and urogenital strains. The former included indels at nt 529 and 138 with TrpA truncation at 184 aa and 46 aa, respectively, and no evidence for B strain operons as previously reported (25). Novel SNPs produced a stop codon in B_HAR36, a frameshift and early truncation in B_QH111L, and an intact *trp*A sequence for B_TZ1A828, which was remarkable and similar to urogenital strains. Except for B_TZ1A828, these findings were not unexpected in that ocular strains have uniformly evolved toward a loss of operon function.

Seven urogenital strains and a single LGV strain from 2003 to 2012 had unique *trp*A mutations. We previously reported on serial isolates of strain F_SF11 (i.e., F_I-IV) isolated in 2010 that had a 2-aa TrpA extension with decreased function *in vitro* (26). These data were surprising as the operon is thought to be highly conserved and functional among urogenital strains. To further analyze evolution among the strains, we evaluated evidence for selective pressure. Both ocular and urogenital strains were under positive selection with virtually no mutations in the LGV strains, suggesting that these latter strains are under strong stabilizing pressure where the operon has likely become fixed. Indeed, LGV strains are known to be markedly more resistant to IFN-γ inhibition than non-LGV strains. Further segregation of the strains by anatomic site showed that urethral strains were under greater positive selection than vaginal strains. The higher Pi(a)/Pi(s) for these strains compared to the ocular strains suggests a more rapid evolution of beneficial mutations. Interestingly, the former were from populations in Argentina, Italy, Russia, Sweden, the United Kingdom, and the United States with diverse genotypes (e.g., D, E, F, G, H, J, and K) and include populations of men who have sex with men (MSM). The high frequency of sexual activity with risky sexual behavior may drive urethral strain selection and mutations in these latter populations (3, 43–45). Selection could also be enhanced by additional pressure from host immune responses elicited by exposure to microbes in various anatomic sites that might have been visited during sex (e.g., oropharynx, rectum). This would indicate that diversification might be favored toward increasing gene fitness (46), but could also lead to deleterious mutations as in Muller’s ratchet (47).

To further explore operon functionality of the mutations, we used a three-dimensional (3D) protein homology modeling approach to assess αββα structural variations as we previously described (26) in addition to TrpR modeling and the effect on protein stability, ligand-binding affinity, and TrpR affinity for ssDNA measured by calculating free energy changes (ΔΔ*G*) between reference and mutant operon proteins. Similar to many other Gram-negative bacteria, expression of chlamydial *trp*BA genes is tightly regulated by TrpR. When in contact with its corepressor tryptophan, TrpR prevents *trp*BA transcription by binding to the *trp* operator-DNA (48). Tryptophan starvation, in contrast, induces transcription of *trp*BA to restore tryptophan levels in the intracellular environment (25, 26). Several studies with E. coli TrpR have reported that indole derivatives such as IAC derepress the operon by forming a “pseudorepressor” that displaces tryptophan and has poor affinity for the operator-DNA, thereby allowing *trp*BA transcription (30, 49, 50). However, if indole is not available as a substrate for synthesis when TS has been produced, ammonia will be produced that is detrimental to *Ct* as it has antibacterial properties (26, 38). This has also been shown to be the case with other intracellular pathogenic bacteria such as Legionella pneumophila and M. tuberculosis (51, 52).

We modeled TrpR mutants and their *Ct* reference strains against the closest homology template, E. coli 6eni. Strains E_UK769748, F_SwabB8, J_SF5, and J_SF6 exhibited a decreased affinity for ligand IAC, and E_UK769748, E_R27091, E_R29005, and F_SwabB8 had increased affinity for ssDNA (*trp* operator-DNA). Since binding of TrpR at the operator-DNA interferes with RNA polymerase binding, initiation of *trp*BA transcription would be prevented (53), eliminating any risk of ammonia production if indole was in low supply. All but E_UK769748 had a net decrease in protein stability with a reduced energy expenditure. Finally, G81V and P84S in E_UK769748 and S35G in J_SF5 had near-neutral effects on protein stability and entropy, suggesting that these mutations arise at no/low fitness cost.

The closest protein model for *Ct* TrpB mutants and their reference strains was E. coli 5ey5 with cofactor PLP and Na^+^ as the ligand. In TS catalysis and regulation, cation activation is achieved through an allosteric linkage connecting the PLP active site (54). MVC consists of a backbone of carbonyls that can accommodate cations of different size and incorporate ligated water molecules to satisfy electrostatic requirements of cation-bound MVC. MVC-bound Na^+^, in contrast to the MVC-free form (i.e., without ion binding), creates a conformational change in the β-COMM domain allowing allosteric interactions with α-L6 in TrpA to a closed conformation with indole tunnel formation. E. coli and hence *Ct* can bind only Na^+^. However, *in vitro* studies of other bacteria have found that cations such as Cs^+^ more effectively induce a closed conformation that enhances TS function compared to Na^+^ (54), indicating that *Ct* strains likely do not have an optimally functional TS.

Urogenital strain D_NL12, E_SotonE8 and I_UK913341 mutations were located within the β-COMM domain and predicted to reduce Na^+^ ligand affinity and therefore TS activity as a result of no/low catalytic activation and perturbed allosteric interactions. βP304S—a nonpolar hydrophobic proline substituted for a polar hydrophilic serine—in the MVC-binding site of E_SotonE8 may have also impacted catalysis and allosteric interactions. Interestingly, mutations D326E in D_SF12, T177K in I_UK913341, and A217V in J_S3107 had increased affinity to PLP, which appeared to be detrimental to the Na^+^ interaction with MVC (Table 2). All of these mutations resulted in decreased protein stability due to the reduced affinity to Na^+^ with reduced energy expenditures.

The TrpA protein model for *Ct* mutants and reference strains was M. tuberculosis 5tch with MLI and FMT as the ligands. G_UK750369 αC177Y and I_UK913341 αE178Q mutations located within α-L6 and G_UK750369 αR206K in the catalytic and subunit interaction domain exhibited reduced ligand affinity for both with reduced protein stability and energy expenditure. Only the αQ37R mutation in G_UK750369 and I_UK913341 predicted an increased protein stability with a lower energy cost, suggesting greater fitness (Table 2). Interestingly, the αC177Y mutation was present in many urogenital reference and mutant strains, indicating that urogenital strains in general have reduced protein stability and ligand affinity for MLI and FMT (Fig. 11a). Furthermore, mutations present elsewhere on the α-subunit of E_Fin214, F_Fin219, and J_UK583676 had reduced ligand affinity and protein stability, indicating a reduction in synthase efficiency through suboptimal allosteric interactions. Our previously reported F_SF11 mutant (26) showed a predicted increase in affinity for MLI and FMT but reduced protein stability. This mutation likely increased energy utilization in interacting with MLI and FMT, which would have had a destabilizing effect on protein structure. This is not surprising in that F_SF11 had markedly decreased tryptophan synthesis and lower uptake of tryptophan for metabolism *in vitro* (26). While it would have been advantageous to perform *in vitro* studies on additional strains in this data set, the majority were not available or not available as viable organisms.

It has been proposed that *Ct* evolved from an ancestor that colonized the gastrointestinal tract (55), a site with abundant indole from indole-producing bacteria. Consistent with this, we found that rectal strains belonging to the LGV and urogenital lineages had intact functional operons while mutations that render the operon nonfunctional were found only among urethral and vaginal/endocervical strains, suggesting that the former strains have reached a high degree of fitness in this anatomic site. *trp*R, *trp*A, and *trp*B have undergone multiple mutation events in the latter strains, indicating disparate evolutionary strategies to either maintain the functional operon or lose function while preserving its ability to scavenge the intracellular host environment for tryptophan. Our findings dispel the dogma in the field that there is strong selective pressure for all urogenital strains to maintain a functional operon. Niche specificity appears to dictate maintenance or loss of function. While the ability to utilize indole seems advantageous for this bacterium, loss of function through evolution and selective pressure as in the case of ocular strains seems to have benefited the organism by allowing it not to have to synthesize tryptophan, which is energetically costly (56). Clinical benefit for this loss of function was also demonstrated in our previous study for sexually transmitted urogenital strain F_SF11 (26). Based on the present study, we are seeing this same trend in urogenital strains across the globe: strains with mutant proteins were inclined toward energy conservation by exhibiting a decreased affinity for their respective ligands. This is not surprising because l-tryptophan is the one of the most energetically costly amino acids to synthesize (56). Our data, therefore, indicate a novel host-pathogen evolutionary mechanism for intracellular survival whereby urogenital strains are evolving more rapidly with mutations that impact tryptophan operon function in a manner that is energetically beneficial for the organism.

## MATERIALS AND METHODS

### Tryptophan operon sequences, genome assembly, and/or operon sequencing.

Of 562 genomes, 486 were available from NCBI SRA and 76 from GenBank. Three unpublished *Ct* genomes and 30 unpublished *Ct* clinical samples with complete operon sequences from the Dean Laboratory were also available, totaling 595 high-quality operon sequences (Fig. 1; see also Table S1 in the supplemental material).

The 486 SRA genomes were imported and assembled in Geneious Prime 2019.2.1 (57); the 21 *Ct* reference genomes were used for mapping SRA reads using the built-in Geneious assembler at low sensitivity/fastest setting with up to 5 iterations. The 76 nearly complete and three unpublished *Ct* genomes were also imported into Geneious. Tryptophan operons consisting of *trp*R, intergenic region (IGR), *trp*B, and *trp*A, in that order, were extracted in Geneious after checking operon mapped reads that were at a minimum coverage depth of 10×. In addition, operons for the 30 clinical samples were sequenced using techniques and primers as previously described (26).

### Alignment, phylogeny, recombination, and mutation analysis.

The operons from 595 reference and clinical samples were concatenated and aligned using MAFFT v7.450 (58). The alignment for each sample was used to construct an approximate maximum likelihood phylogenetic tree using FastTree 2.1.11 (59) with a generalized time-reversible model. Both MAFFT and FastTree were built-in and executed in Geneious; 1,000-bootstrap sampling was used for statistical support of the tree. The tree together with the metadata on *omp*A genotype and anatomic site of infection was used to generate the phylogenetic tree in Tree of Life v5.5 (60).

The MAFFT alignment of the operon sequences was also used to detect recombination events by RDP (61), GENECONV (62), BOOTSCAN (63), MAXCHI (64), CHIMAERA (65), SISCAN (66), PHYLPRO (67), LARD (68), and 3Seq (69). All software was built-in and executed in RDP4. The full exploratory scan was performed using each of the methods followed by a scan using the individual software. Additionally, a secondary recombination analysis was performed on putative recombinants identified from the RDP4 analysis to further tease out the breakpoint analysis of any recombination events noted. Briefly, nucleotide MAFFT alignments of the putative recombinants and their potential parents were submitted to Recco (70).

Operon sequences from the 21 reference strains (A_HAR13, B_HAR36, Ba_Apache2, C_TW3OT, D_UW3Cx, Da_TW448, E_Bour, F_IC-Cal13, G_UW57Cx, H_UW4Cx, I_UW12Ur, Ia_UW202, J_UW36Cx, Ja_UW92, K_UW31Cx, L_1__440, L_2__434, L_2_a_UW396, L_2_b_UCH1proctitis, L_2_c, and L_3__404) were aligned using MAFFT to build a phylogenetic tree (Fig. S1). These data were used to determine *omp*A genotype and generate SNPs in the Geneious variant finder for the 595 genome sequences. Amino acid sequences with substitutions, premature stop codons, extensions, or frameshifts compared to respective reference sequences were designated mutants; their functionality was further studied by three-dimensional (3D) homology and functional protein modeling (see below).

DnaSP 6.0 (71) was used to analyze operon sequence polymorphisms to determine evidence for selection by calculating the ratio of nonsynonymous to synonymous substitutions [Pi(a)/Pi(s)] (Jukes-Cantor corrected), number of polymorphic (segregating) sites, and haplotype diversity.

### 3D protein structure homology modeling of *Ct* operon mutant and reference strains.

TrpR, TrpB, and TrpA protein structure homology modeling was performed as previously described (26) with few modifications. Briefly, *Ct* mutant and reference operon protein sequences were uploaded to MODBASE, and MODELLER (31, 72) was used to calculate comparative 3D protein structures using PSI-BLAST (73), pairwise sequence alignment, sequence-sequence, and sequence-profile. PyMOL was used for structural visualization and imaging (The PyMOL Molecular Graphics System, version 1.2r3pre; Schrödinger, LLC). Predictions from MODELLER for both structure and functional residues were verified using the Simple Modular Architecture Research Tool (SMART) (74), SignalIP (75), Protein family (Pfam) (76), Conserved Domain (CD) (77), and Transmembrane helix prediction (TMHMM) (78). With a cutoff of 0.01 E value to perform protein BLAST against the Protein Data Bank (PDB) in addition to Phyre (Protein Homology/analogy Recognition Engine) (79), we searched for other homologs for structuring the tryptophan operon proteins.

Predicted models were used to manually identify and visualize nonsynonymous mutations, TrpR ligand-binding sites (LBSs), and DNA-binding motifs (DBM), TrpA α-Loop 6 (α-L6) and catalytic site, and TrpB beta communication (β-COMM) domain and monovalent cation (MVC)-binding sites, and indole tunnel based on both our prior research and crystal structures of respective bacterial strains (24, 30).

### Molecular docking, binding site analysis, and effect of mutations on TrpR, TrpB and TrpA protein structure stability and ligand-binding affinity.

Molecular docking and binding site analyses were performed using AutoDock (80) and PyMOL (81). AutoDock performs automated docking by simulated annealing, local gradient search, and genetic algorithm. Final atomic conformations were the result of minimizing the energy in a force field using multiple possible schemes. The combination of a genetic algorithm with inheritance of local optimizations yielded a Lamarckian genetic algorithm.

Ligand structural information was obtained from PubChem (82) where 3D structures were drawn in Chimera (83); geometry was optimized using the steepest descent method followed by conjugate gradient algorithms. Calculations with 50 intervals were used to ensure the highest accuracy of energy optimization. Since ligands are small molecules, the Gasteiger force field was assigned for the calculations.

Docking involved the prediction of ligand conformation and orientation within a targeted binding site. The Gibbs free energy correlation derived from molecular conformations was obtained using AutoDock. The free energy of binding (Δ*G*) is related to binding affinity and the equilibrium constant in the equation Δ*G* = −RT ln *K_A_*.

Modeling the *Ct* operon mutations was performed as previously described (84) with modifications. To predict the effect of mutations on protein-structure stability, ssDNA affinity, and ligand affinity binding, mCSM (http://biosig.unimelb.edu.au/mcsm/stability), mCSM-NA (http://biosig.unimelb.edu.au/mcsm_na/prediction), and mCSM-lig (http://biosig.unimelb.edu.au/mcsm_lig/) were used. PDB files of *Ct* reference protein templates and text files containing sets of mutations in mutant strains were provided as inputs to the respective program to estimate the free energy difference (ΔΔ*G*) between reference and mutant protein forms as a measure of thermodynamic stability and estimate the affinity change of small-molecule ligands for proteins as log-affinity fold change. The PyMOL plugin Arpeggio (http://biosig.unimelb.edu.au/arpeggioweb/) was used to study and visualize interatomic interactions.
