# Supplementary material for: Tryptophan Operon Diversity Reveals Evolutionary Trends among Geographically Disparate Chlamydia trachomatis Ocular and Urogenital Strains Affecting Tryptophan Repressor and Synthase Function
Source: mBio. 2021 May 11;12(3):e00605-21. doi: 10.1128/mBio.00605-21 (PMC8262981; doi:10.1128/mBio.00605-21)
Supplement: TABLE S1 [file mbio.00605-21-st001.pdf]

Table S1. Primary recombination analysis in the tryptophan operon for global *C. trachomatis* strains (n=595) using RDP4.

	Daughter	Major parent	Minor parent	Beginning	Ending	Uncorrected	MC corrected	Identified by (Av. P-Val)	Recombinant score
1	**B_9**	A_HAR13	L3_404LN	1090	1450	$6.163X10^{-10}$	$7.396X10^{-8}$	GENECONV ($1.123X10^{-2}$)<br>Bootscan ($1.124X10^{-3}$)<br>Maxchi ($2.381X10^{-4}$)<br>3Seq ($3.223X10^{-5}$)	0.549 (B_9)<br>0.196 (A_HAR13)<br>0.255 (L3_404LN)
2	**A_8**	A_HAR13	L3_404LN	971	1555	$8.421X10^{-6}$	$1.011X10^{-3}$	GENECONV ($1.123X10^{-2}$)<br>Bootscan ($1.124X10^{-3}$)<br>Maxchi ($2.381X10^{-4}$)<br>3Seq ($3.223X10^{-5}$)	0.549 (B_9)<br>0.196 (A_HAR13)<br>0.255 (L3_404LN)
		A_HAR13	B_9	971	1555	$8.431X10^{-6}$	$1.011X10^{-3}$	MaxChi ($2.629X10^{-3}$)<br>3Seq ($1.360X10^{-2}$)	0.549 (B_9)<br>0.196 (A_HAR13)<br>0.255 (L3_404LN)
	**A_15**	A_HAR13<br>(99.8%)	L3_404LN	1012	1438	$5.575X10^{-8}$	$6.908X10^{-6}$	GENECONV ($1.123X10^{-2}$)<br>Bootscan ($1.124X10^{-3}$)<br>Maxchi ($2.381X10^{-4}$)<br>3Seq ($3.223X10^{-5}$)	0.549 (B_9)<br>0.196 (A_HAR13)<br>0.255 (L3_404LN)
		A_HAR13<br>(99.8%)	B_9 (92.9%)	1090	1206	$5.604X10^{-5}$	$6.725X10^{-3}$	MaxChi ($2.629X10^{-3}$)<br>3Seq ($1.360X10^{-2}$)	0.446 (A_15)<br>0.215 (A_HAR13)<br>0.339 (B_9)
	**A_S45**	A_HAR13	B_9 (99.6%)	1339	1206	$1.567X10^{-4}$	$1.881X10^{-2}$	MaxChi ($2.629X10^{-3}$)<br>3Seq ($1.360 X10^{-2}$)	0.446 (A_S45)<br>0.215 (A_HAR13)<br>0.339 (B_9)
3	**B_QH111L**	D_Fin163 (99.3%)	E_UK769748	332	1912	$1.182X10^{-4}$	$1.418X10^{-2}$	3Seq ($1.418X10^{-2}$)	0.452 (B_QH111L)<br>0.305 (D_Fin163)<br>0.242 (E_UK769748)
4	**I_UK913341**	L3_404LN<br>(99.3%)	E_UK769748	433	2486	$4.014X10^{-4}$	$4.817X10^{-2}$	3Seq ($7.766X10^{-1}$)	0.537 (I_UK913341)<br>0.253 (L3_404LN)<br>0.210 (E_UK769748)
	**Ia_SF16**	L3_404LN<br>(98.9%)	E_UK769748 (99.8%)	433	2486	$7.87 X 10^{-3}$	0.944	3Seq ($7.766X10^{-1}$)	0.537 (Ia_SF16)<br>0.253 (L3404LN)<br>0.210 (EUK769748)

**Ia_SF27**	L3_404LN (98.9%)	E_UK769748 (99.8%)	433	2486	7.87×10^{-3}	0.944	3Seq (7.766×10^{-1})	0.537 (Ia_SF27)<br>0.253 (L3_404LN)<br>0.210 (E_UK769748)
**I_NL58**	L3_404LN (98.9%)	E_UK769748 (99.8%)	433	2486	6.597×10^{-3}	0.791	3Seq (7.766×10^{-1})	0.537 (I_NL58)<br>0.253 (L3_404LN)<br>0.210 (E_UK769748)
**I_NL63**	L3_404LN (98.9%)	E_UK769748 (99.8%)	433	2486	6.597×10^{-3}	0.791	3Seq (7.766×10^{-1})	0.537 (I_NL63)<br>0.253 (L3_404LN)<br>0.210 (E_UK769748)
**I_NL69**	L3_404LN (98.9%)	E_UK769748 (99.8%)	433	2486	6.597×10^{-3}	0.791	3Seq (7.766×10^{-1})	0.537 (I_NL69)<br>0.253 (L3_404LN)<br>0.210 (E_UK769748)
**I_NL70**	L3_404LN (98.9%)	E_UK769748 (99.8%)	433	2486	6.597×10^{-3}	0.791	3Seq (7.766×10^{-1})	0.537 (I_NL70)<br>0.253 (L3_404LN)<br>0.210 (E_UK769748)
**I_S2459**	L3_404LN (98.9%)	E_UK769748 (99.8%)	433	2486	6.597×10^{-3}	0.791	3Seq (7.766×10^{-1})	0.537 (I_S2459)<br>0.253 (L3_404LN)<br>0.210 (E_UK769748)
**Ia_20-97**	L3_404LN (98.9%)	E_UK769748 (99.8%)	433	2486	6.597×10^{-3}	0.791	3Seq (7.766×10^{-1})	0.537 (Ia_20-97)<br>0.253 (L3_404LN)<br>0.210 (E_UK769748)
**Ia_Soton1**	L3_404LN (98.9%)	E_UK769748 (99.8%)	433	2486	6.597×10^{-3}	0.791	3Seq (7.766×10^{-1})	0.537 (Ia_Soton1)<br>0.253 (L3_404LN)<br>0.210 (E_UK769748)
**Ia_Soton3**	L3_404LN (98.9%)	E_UK769748 (99.8%)	433	2486	6.597×10^{-3}	0.791	3Seq (7.766×10^{-1})	0.537 (Ia_Soton2)<br>0.253 (L3_404LN)<br>0.210 (E_UK769748)
**Ia_UW202**	L3_404LN (98.9%)	E_UK769748 (99.8%)	433	2486	8.33×10^{-3}	1	3Seq (7.766×10^{-1})	0.537 (Ia_UW202)<br>0.253 (L3_404LN)<br>0.210 (E_UK769748)
