# Supplementary material for: Tryptophan Operon Diversity Reveals Evolutionary Trends among Geographically Disparate Chlamydia trachomatis Ocular and Urogenital Strains Affecting Tryptophan Repressor and Synthase Function
Source: mBio. 2021 May 11;12(3):e00605-21. doi: 10.1128/mBio.00605-21 (PMC8262981; doi:10.1128/mBio.00605-21)
Supplement: DATA SET S1 [file mbio.00605-21-sd001.pdf]

Supplementary Data 1. List of *C. trachomatis* strains used in this study and associated metadata for the tryptophan operon nucleotide sequence.

Name	Lineage	*omp* A Genotyp	Country	Gender	Year	Source	operon (bp)	ENA ERR	ENA ERS	NCBI	Study accession	Genome Reference
A_A8	ocular	A	Sudan	M	2016-2019	ocular	2563				PRJEB32246	Alkidir 2019
A_A15	ocular	A	Sudan	F	2016-2019	ocular	2563				PRJEB32246	Alkidir 2019
A_363	ocular	A	Tanzania	F	2000	ocular	2565	ERR034213	ERS017900	-		Harris 2012
A_2497	ocular	A	Tanzania	F	2000	ocular	2565	-	-	FM872306		Harris 2012
A_5291	ocular	A	Tanzania	F	2000	ocular	2565	ERR034214	ERS017901	-		Harris 2012
A_7249	ocular	A	Tanzania	M	2000	ocular	2565	ERR034215	ERS017902	-		Harris 2012
A_D213	ocular	A	Gambia	M	2001	ocular	2562	ERR175652	ERS177838	-		
A_D230	ocular	A	Gambia	F	2001	ocular	2562	ERR111554	ERS075177	-		
A_HAR13	ocular	A	Egypt	F	1958	conjunctiva	2356	-	-	CP000051		Carlson 2005
A_MH858	ocular	A	Tanzania	M	2000	ocular	2565	ERR175560	ERS177716	-		
A_MH1364	ocular	A	Tanzania	F	2000	ocular	2565	ERR175575	ERS177731	-		
A_MH2145	ocular	A	Tanzania	F	2000	ocular	2565	ERR175561	ERS177717	-		
A_MH2497	ocular	A	Tanzania	F	2000	ocular	2565	ERR175562	ERS177718	-		
A_MH3234	ocular	A	Tanzania	F	2000	ocular	2565	ERR175576	ERS177732	-		
A_MH4510	ocular	A	Tanzania	F	2000	ocular	2565	ERR175577	ERS177733	-		
A_MH5368	ocular	A	Tanzania	F	2000	ocular	2565	ERR175563	ERS177719	-		
A_MH5786	ocular	A	Tanzania	M	2000	ocular	2565	ERR175564	ERS177720	-		
A_MH6446	ocular	A	Tanzania	M	2000	ocular	2565	ERR175578	ERS177734	-		
A_MH7205	ocular	A	Tanzania	M	2000	ocular	2565	ERR175579	ERS177735	-		
A_MH8910	ocular	A	Tanzania	M	2000	ocular	2565	ERR175580	ERS177736	-		
A_MH9922	ocular	A	Tanzania	F	2000	ocular	2565	ERR175581	ERS177737	-		
A_MH10549	ocular	A	Tanzania	M	2000	ocular	2565	ERR175582	ERS177738	-		
A_MH10648	ocular	A	Tanzania	F	2000	ocular	2565	ERR175583	ERS177739	-		
A_MH10901	ocular	A	Tanzania	M	2000	ocular	2565	ERR175584	ERS177740	-		
A_MH11715	ocular	A	Tanzania	M	2000	ocular	2565	ERR175585	ERS177741	-		
A_MH11979	ocular	A	Tanzania	M	2000	ocular	2565	ERR175586	ERS177742	-		
A_MH12023	ocular	A	Tanzania	F	2000	ocular	2565	ERR175565	ERS177721	-		
A_MH13849	ocular	A	Tanzania	M	2000	ocular	2565	ERR175566	ERS177722	-		
A_MH14553	ocular	A	Tanzania	F	2000	ocular	2565	ERR175567	ERS177723	-		
A_MH15048	ocular	A	Tanzania	F	2000	ocular	2565	ERR175587	ERS177743	-		
A_MH15741	ocular	A	Tanzania	F	2000	ocular	2565	ERR175588	ERS177744	-		
A_MH16005	ocular	A	Tanzania	M	2000	ocular	2565	ERR175568	ERS177724	-		
A_MH16170	ocular	A	Tanzania	M	2000	ocular	2565	ERR175589	ERS177745	-		
A_MH16665	ocular	A	Tanzania	M	2000	ocular	2565	ERR175590	ERS177746	-		
A_MH17127	ocular	A	Tanzania	M	2000	ocular	2565	ERR175591	ERS177747	-		
A_MH18843	ocular	A	Tanzania	F	2000	ocular	2565	ERR175569	ERS177725	-		
A_MH18876	ocular	A	Tanzania	F	2000	ocular	2565	ERR175592	ERS177748	-		
A_MH19657	ocular	A	Tanzania	M	2000	ocular	2565	ERR175570	ERS177726	-		

A_MH19679	ocular	A	Tanzania	F	2000	ocular	2565	ERR175593	ERS177749	-		
A_MH20130	ocular	A	Tanzania	F	2000	ocular	2565	ERR175594	ERS177750	-		
A_MH20933	ocular	A	Tanzania	F	2000	ocular	2565	ERR175595	ERS177751	-		
A_MH21571	ocular	A	Tanzania	F	2000	ocular	2565	ERR175596	ERS177752	-		
A_MH23527	ocular	A	Tanzania	F	2000	ocular	2565	ERR175571	ERS177727	-		
A_MH24519	ocular	A	Tanzania	M	2000	ocular	2565	ERR175597	ERS177753	-		
A_MH24640	ocular	A	Tanzania	F	2000	ocular	2565	ERR175598	ERS177754	-		
A_MH24673	ocular	A	Tanzania	M	2000	ocular	2565	ERR175572	ERS177728	-		
A_MH25256	ocular	A	Tanzania	M	2000	ocular	2565	ERR175573	ERS177729	-		
A_MH25883	ocular	A	Tanzania	F	2000	ocular	2565	ERR175599	ERS177755	-		
A_MH26862	ocular	A	Tanzania	F	2000	ocular	2565	ERR175600	ERS177756	-		
A_MH27137	ocular	A	Tanzania	F	2000	ocular	2565	ERR175601	ERS177757	-		
A_MH35739	ocular	A	Tanzania	M	2000	ocular	2565	ERR175603	ERS177759	-		
A_MH47300	ocular	A	Tanzania	F	2000	ocular	2565	ERR175574	ERS177730	-		
A_MH53658	ocular	A	Tanzania	F	2000	ocular	2565	ERR175604	ERS177760	-		
A_SA1	ocular	A	Saudi Arabia	F	1957	ocular	2562	ERR558498	ERS177777	-		
A_SB002739	ocular	A	Solomon Island		2013	ocular	2562	-	-	CP016418		Butcher 2016
A_SB006930	ocular	A	Solomon Island		2013	ocular	2562	-	-	CP016420		Butcher 2016
A_SB008107	ocular	A	Solomon Island		2013	ocular	2562	-	-	CP016422		Butcher 2016
A_SB013112	ocular	A	Solomon Island		2013	ocular	2562	-	-	CP016424		Butcher 2016
A_SB013321	ocular	A	Solomon Island		2013	ocular	2562	-	-	CP016426		Butcher 2016
A_B1	ocular	A	Sudan	M	2016-2019	ocular	2562			-	PRJEB32246	Alkidir 2019
A_B9	ocular	A	Sudan	F	2016-2019	ocular	2563			-	PRJEB32246	Alkidir 2019
A_B13	ocular	A	Sudan	F	2016-2019	ocular	2563			-	PRJEB32246	Alkidir 2019
A_S45	ocular	A	Sudan	M	2016-2019	ocular	2563			-	PRJEB32246	Alkidir 2019
A_S59	ocular	A	Sudan	F	2016-2019	ocular	2562			-	PRJEB32246	Alkidir 2019
A_T94	ocular	A	Sudan	F	2016-2019	ocular	2562			-	PRJEB32246	Alkidir 2019
A_TF16	ocular	A	Sudan	F	2016-2019	ocular	2562			-	PRJEB32246	Alkidir 2019
A_TF34	ocular	A	Sudan	F	2016-2019	ocular	2562			-	PRJEB32246	Alkidir 2019
A_TF54	ocular	A	Sudan	M	2016-2019	ocular	2562			-	PRJEB32246	Alkidir 2019
A_J52	ocular	A	Sudan	M	2016-2019	ocular	2562			-	PRJEB32246	Alkidir 2019
Ba_Apache2	ocular	Ba	USA		1960	ocular	2563	ERR140762	ERS095032	-		
B_Aus2	genital	B-Ba	Australia			unknown	2566	ERR189742	ERS153019	-		Andersson 2016
B_Aus3	genital	B-Ba	Australia		1986-1989	unknown	2566	ERR189743	ERS153020	-		Andersson 2016
B_Aus4	genital	B-Ba	Australia		1986-1989	unknown	2566	ERR189744	ERS153021	-		Andersson 2016
B_Aus5	genital	B-Ba	Australia		1986-1989	unknown	2566	ERR189745	ERS153022	-		Andersson 2016
B_Aus6	genital	B-Ba	Australia		1986-1989	unknown	2566	ERR189766	ERS153043	-		Andersson 2016
Ba_Aus25	genital	Ba	Australia		1988	ocular	2566	ERR386232	ERS351392	-		Andersson 2016
Ba_Aus28	genital	Ba	Australia		1988	ocular	2566	ERR386222	ERS351377	-		Andersson 2016
B_Aus36	genital	B-Ba	Australia		1989	ocular	2566	ERR386225	ERS351385	-		Andersson 2016
B_Aus40	genital	B-Ba	Australia			endocervic	2566	ERR999730	ERS747489	-		Andersson 2016

B_Aus41	genital	B-Ba	Australia			endocervic	2566	ERR999731	ERS747490	-	Andersson 2016
B_Aus42	genital	B-Ba	Australia			endocervic	2566	ERR999732	ERS747491	-	Andersson 2016
B_Aus43	genital	B-Ba	Australia			endocervic	2566	ERR999733	ERS747492	-	Andersson 2016
B_Aus44	genital	B-Ba	Australia			endocervic	2566	ERR999734	ERS747493	-	Andersson 2016
B_Aus45	genital	B-Ba	Australia			endocervic	2566	ERR999735	ERS747494	-	Andersson 2016
B_Fin101	genital	B-Ba	Finland	F	2009	endocervix	2566	ERR278185	ERS200114	-	
B_Fin203	genital	B-Ba	Finland	F	2011	endocervix	2566	ERR278154	ERS200083	-	
B_HAR36	ocular	B-Ba	Saudi Arabia		1969	ocular	2562	ERR189736	ERS153013	-	
B_Jali16	ocular	B-Ba	Gambia		1985	ocular	2562	ERR189738	ERS153015	-	
B_Jali20	ocular	B-Ba	Gambia		1985	ocular	2562	-	-	FM872308	Seth-Smith 2009
B_M48	ocular	B-Ba	Gambia	M	2007	ocular	2564	ERR175631	ERS177817	-	
B_NL2	genital	B-Ba	Netherlands	F	2001	endocervix	2566	ERR211016	ERS161066	-	
B/QH111L		B	China	M	2016	ocular	2434			CP018052	Feng Le 2016
B_Sou42	genital	B-Ba	UK		1985	Unknown	2566	ERR210997	ERS161047	-	
B_TZ1A828	ocular	B-Ba	Tanzania	F	1998	ocular	2541	-	-	FM872307	Seth-Smith 2009
C_Aus8	genital	C	Australia		1986-1989	unknown	2566	ERR210992	ERS161042	-	Andersson 2016
C_Aus9	genital	C	Australia		1986-1989	unknown	2566	ERR189768	ERS153045	-	Andersson 2016
C_Aus10	genital	C	Australia		1986-1989	unknown	2566	ERR189746	ERS153023	-	Andersson 2016
C_Aus30	genital	C	Australia	F	1988	ocular	2566	ERR386223	ERS351383	-	Andersson 2016
C_Aus33	genital	C	Australia	M	1988	ocular	2566	ERR386224	ERS351384	-	Andersson 2016
C_TW3	ocular	C	Taiwan		1959	ocular	2562	-	-	CP006945	
C_UW10	ocular	C	Canada	M	1964	ocular	2562	ERR175630	ERS177816	-	-
Da_TW448	ocular	Da	Taiwan		1985	Conjunctiv	2562				This study
D/13-96		D	USA, Seattle	NA	1996	endocervix		-	-	CP006676	Putman 2013
D/14-96		D	USA, Seattle	NA	1996	endocervix		-	-	CP006677	Putman 2013
D_Aus11	genital	D	Australia		1986-1989	unknown	2566	ERR189747	ERS153024	-	Andersson 2016
D_Aus12	genital	D	Australia		1986-1989	unknown	2566	ERR189767	ERS153044	-	Andersson 2016
D_C32	genital	D	UK	F	2011	Cx/Urethra	2566	ERR175621	ERS177807	-	-
D/CS637/11		D		F		endocervix				CP007131	Borges 2015
D_Fin163	genital	D	Finland	F	2010	endocervix	2566	ERR278143	ERS200072	-	-
D_Fin178	genital	D	Finland	F	2010	endocervix	2566	ERR278147	ERS200076	-	-
D_Fin187	genital	D	Finland	F	2010	endocervix	2566	ERR278150	ERS200079	-	-
D_HonLC4	genital	D	Honduras			unknown	2566	ERR658366	ERS151260	-	-
D_HPA314	genital	D	UK	F		endocervix	2566	ERR108297	ERS082979	-	-
D_NL4	genital	D	Netherlands	F	2001	endocervix	2566	ERR211017	ERS161067	-	-
D_NL5	genital	D	Netherlands	F	2001	endocervix	2566	ERR211018	ERS161068	-	-
D_NL6	genital	D	Netherlands	F	2001	endocervix	2566	ERR164672	ERS133259	-	-
D_NL8	genital	D	Netherlands	F	2001	endocervix	2566	ERR164674	ERS133261	-	-
D_NL10	genital	D	Netherlands	F	2001	endocervix	2566	ERR164676	ERS133263	-	-
D_NL11	genital	D	Netherlands	F	2001	endocervix	2566	ERR164677	ERS133264	-	-
D_NL12	genital	D	Netherlands	F	2001	endocervix	2566	ERR164678	ERS133265	-	-

D_NL13	genital	D	Netherlands	F	2001	endocervix	2566	ERR164679	ERS133266	-	-
D_NL14	genital	D	Netherlands	F	2001	endocervix	2566	ERR164680	ERS133267	-	-
D_NL15	genital	D	Netherlands	F	2001	endocervix	2566	ERR164681	ERS133268	-	-
D_NL16	genital	D	Netherlands	F	2001	endocervix	2566	ERR210970	ERS161020	-	-
D_NL17	genital	D	Netherlands	F	2001	endocervix	2566	ERR164682	ERS133269	-	-
D_NL19	genital	D	Netherlands	F	2001	endocervix	2566	ERR164684	ERS133271	-	-
D_NL32	genital	D	Netherlands	F	2001	endocervix	2566	ERR189763	ERS153040	-	-
D_NL59	genital	D	Netherlands	F	2001	endocervix	2566	ERR189765	ERS153042	-	-
D_NL71	genital	D	Netherlands	F	2001	endocervix	2566	ERR210988	ERS161038	-	-
D_S276I	genital	D	Sweden	F	2011	endocervix	2566	ERR140754	ERS095024	-	-
D_S1736	genital	D	Sweden	F	2010	endocervix	2566	ERR108286	ERS082968	-	-
D_S1879	genital	D	Sweden	M	2010	urethra	2566	ERR108287	ERS082969	-	-
D_S2130	genital	D	Sweden	F	2010	endocervix	2566	ERR111586	ERS075209	-	-
D_S3257	genital	D	Sweden	F	2010	endocervix	2566	ERR111590	ERS075213	-	-
D_S3489	genital	D	Sweden	F	2010	endocervix	2566	ERR111589	ERS075212	-	-
D_S3929	genital	D	Sweden	F	2010	endocervix	2566	ERR111578	ERS075201	-	-
D_S4093	genital	D	Sweden	F	2010	endocervix	2566	ERR140804	ERS095074	-	-
D_S4828	genital	D	Sweden	F	2010	endocervix	2566	ERR108294	ERS082976	-	-
D_SF2	genital	D	USA	F	2003	endocervix	2566				This study
D_SF12	genital	D	USA	F	2003	endocervix	2566				This study
D_Soton15	genital	D	UK	F	2009	endocervix	2566	ERR026568	ERS013807	-	-
D_Soton42	genital	D	UK	F	2009	endocervix	2566	ERR140819	ERS095089	-	-
D_Soton47	genital	D	UK	F	2009	endocervix	2566	ERR026573	ERS013812	-	-
D_Soton49	genital	D	UK	F	2009	endocervix	2566	ERR140821	ERS095091	-	-
D_Soton54	genital	D	UK	F	2009	endocervix	2566	ERR140822	ERS095092	-	-
D_Soton128	genital	D	UK	F	2009	endocervix	2566	ERR140826	ERS095096	-	-
D_Soton150	genital	D	UK	F	2009	endocervix	2566	ERR026586	ERS013823	-	-
D_SotonD1	genital	D	UK	F	2009	endocervix	2566	ERR027327	ERS008761	-	Harris 2012
D_SotonD2	genital	D	UK	F	2009	endocervix	2566	ERR026542	ERS013784	-	
D_SotonD3	genital	D	UK	F	2009	endocervix	2566	ERR026545	ERS013785	-	
D_SotonD4	genital	D	UK	F	2009	endocervix	2566	ERR026546	ERS013786	-	
D_SotonD5	genital	D	UK	F	2009	endocervix	2566	ERR026547	ERS013787	-	Harris 2012
D_SotonD6	genital	D	UK	F	2009	endocervix	2566	ERR027328	ERS008762	-	Harris 2012
D_SQ29	genital		USA		1991	unknown	2566			CP017731	
D_SQ32	genital		USA		1995	unknown	2566			CP017730	
D_STN101	genital	D	UK		1985	unknown	2566	ERR658584	ERS208565	-	-
D_STN113	genital	D	UK		1985	unknown	2566	ERR658586	ERS208567		
D_T9p	genital	D	UK		2012	unknown	2566	ERR140781	ERS095051	-	-
D_UK466322	genital	D	UK	F	2012	Cx/Urethra	2566	ERR658635	ERS208362	-	-
D_UK663610	genital	D	UK	F	2012	Cx/Urethra	2566	ERR658666	ERS208393	-	-
D_UK750376	genital	D	UK	M	2012	urethra	2566	ERR658498	ERS160257	-	-

D_UK750523	genital	D	UK	F	2012	endocervix	2566	ERR658501	ERS160260	-	-
D_UK912432	genital	D	UK	F	2012	Cx/Urethra	2566	ERR658673	ERS208400	-	-
D_UW3CX	genital	D	USA	F	1965	endocervix	2566	-	-	AE001273	Stephens 1998
E-103	genital	E	Germany	F	1992	unknown	2566			CP015294	Eder 2017
E_150	genital	E	0	M		rectum	2566	-	-	CP001886	Jeffrey 2010
E_160	genital	E	Germany	F	1995	unknown	2566			CP001886	Eder 2017
E_547	genital	E	Germany	F	1991	unknown	2566			CP015298	Eder 2017
E_940U470	genital	E	USA	M		urethra	2566	ERR348845	ERS248053	-	-
E_12-94	genital	E	USA			unknown	2566	-	-	CP006675	Putman 2013
E_8873	genital	E	Germany	F	1998	unknown	2566			CP015300	Eder 2017
E_11023	genital	E		F		endocervix	2566			CP001890	Jeffrey 2010
E_32931	genital	E	Germany	F		vagina	2566			CP015302	Eder 2017
E_Ar5	genital	E	Argentina	F	2006	endocervix	2566	ERR111634	ERS082951	-	-
E_Ar152	genital	E	Argentina	F	2005	ocular	2566	ERR111636	ERS082953	-	-
E_Ar182	genital	E	Argentina	M	2008	urethra	2566	ERR108273	ERS082955	-	-
E_Ar250	genital	E	Argentina	M	2006	ocular	2566	ERR658410	ERS160297		
E_Ar427	genital	E	Argentina	F	2011	endocervix	2566	ERR108279	ERS082961	-	-
E_Ar7218	genital	E	Argentina	M	2004	ocular	2566	ERR108280	ERS082962	-	-
E_Aus13	genital	E	Australia		1986-1989	unknown	2566	ERR189761	ERS153038	-	Andersson 2016
E_Bour	genital	E	USA	M	1959	ocular	2566	ERR008578	ERS001401	HE603212	Harris 2012
E_C5	genital	E	UK	F	2011	vagina	2566	ERR175618	ERS177804	-	-
E_C37	genital	E	UK	F	2011	vagina	2566	ERR175623	ERS177809	-	-
E_C58	genital	E	UK	F	2011	vagina	2566	ERR175626	ERS177812	-	-
E_C194	genital	E	UK	F	2011	vagina	2566	ERR175638	ERS177824	-	-
E_C208	genital	E	UK	M	2011	Urine	2566	ERR175640	ERS177826	-	-
E_C236	genital	E	UK	F	2011	Ur/Cx	2566	ERR175643	ERS177829	-	-
E_C258	genital	E	UK		2011	unknown	2566	ERR175646	ERS177832	-	-
E_C599	genital	E	USA	M	1996	urethra	2566	ERR027326	ERS008760	-	-
E_CC15	genital	E	UK	F	2009	endocervix	2566	ERR140842	ERS095112	-	-
E_CC35	genital	E	UK	M	2010	urethra	2566	ERR140843	ERS095113	-	-
E_CLIN1	genital	E	USA			endocervix	2566				This study
E_CLIN3	genital	E	USA			endocervix	2566				This study
E_CS102511	genital	E	NA	F	2015	endocervix				CP010567	Borges 2015
E_DK20	genital	E	Denmark		1967	conjunctiva	2566	ERR558495	ERS177774	-	-
E_Fin127	genital	E	Finland	F	2010	endocervix	2566	ERR278134	ERS200063	-	-
E_Fin129	genital	E	Finland	F	2010	endocervix	2566	ERR278135	ERS200064	-	-
E_Fin142	genital	E	Finland	F	2010	endocervix	2566	ERR278136	ERS200065	-	-
E_Fin155	genital	E	Finland	F	2010	endocervix	2566	ERR278141	ERS200070	-	-
E_Fin159	genital	E	Finland	F	2010	endocervix	2566	ERR278187	ERS200116	-	-
E_Fin172	genital	E	Finland	F	2010	endocervix	2566	ERR278145	ERS200074	-	-
E_Fin184	genital	E	Finland	F	2010	endocervix	2566	ERR278148	ERS200077	-	-

E_Fin185	genital	E	Finland	F	2010	endocervix	2566	ERR278149	ERS200078	-	-
E_Fin194	genital	E	Finland	F	2010	endocervix	2566	ERR278152	ERS200081	-	-
E_Fin198	genital	E	Finland	F	2010	endocervix	2566	ERR278153	ERS200082	-	-
E_Fin214	genital	E	Finland	F	2011	endocervix	2566	ERR278158	ERS200087	-	-
E_Fin220	genital	E	Finland	F	2011	endocervix	2566	ERR278161	ERS200090	-	-
E_It246	genital	E	Italy	F	2010	endocervix	2566	ERR658535	ERS208511	-	-
E_It363	genital	E	Italy	F	2010	endocervix	2566	ERR658534	ERS208510	-	-
E_It769	genital	E	Italy	F	2011	endocervix	2566	ERR658536	ERS208512	-	-
E_It807	genital	E	Italy			unknown	2566	ERR658407	ERS160294	-	-
E_IU824	genital	E	USA	F		endometria	2566	ERR558500	ERS177779	HF562298	O'Neill 2013
E_IU888	genital	E	USA	F		endometria	2566	ERR558501	ERS177780	HF562300	O'Neill 2013
E_NL21	genital	E	Netherlands	F	2001	endocervix	2566	ERR211019	ERS161069	-	-
E_NL23	genital	E	Netherlands	F	2001	endocervix	2566	ERR189756	ERS153033	-	-
E_NL24	genital	E	Netherlands	F	2001	endocervix	2566	ERR189757	ERS153034	-	-
E_NL26	genital	E	Netherlands	F	2001	endocervix	2566	ERR164687	ERS133274	-	-
E_NL28	genital	E	Netherlands	F	2001	endocervix	2566	ERR164689	ERS133276	-	-
E_NL29	genital	E	Netherlands	F	2001	endocervix	2566	ERR189758	ERS153035	-	-
E_R526	genital	E	Russia	F	2011	endocervix	2566	ERR111568	ERS075191	-	-
E_R1430	genital	E	Russia	M	2011	urethra	2566	ERR111621	ERS082938	-	-
E_R4159	genital	E	Russia	M	2011	urethra	2566	ERR111615	ERS082932	-	-
E_R4195	genital	E	Russia	F	2011	endocervix	2566	ERR140796	ERS095066	-	-
E_R4528	genital	E	Russia	F	2011	endocervix	2566	ERR140835	ERS095105	-	-
E_R16965	genital	E	Russia	F	2011	endocervix	2566	ERR140837	ERS095107	-	-
E_R25114	genital	E	Russia	F	2010	endocervix	2566	ERR111596	ERS075219	-	-
E_R26833	genital	E	Russia	F	2010	endocervix	2566	ERR111601	ERS082918	-	Seth-Smith 2013
E_R27091	genital	E	Russia	F	2010	endocervix	2566	ERR111566	ERS075189	-	Seth-Smith 2013
E_R28017	genital	E	Russia	F	2010	endocervix	2566	ERR111565	ERS075188	-	Seth-Smith 2013
E_R28044	genital	E	Russia	F	2010	endocervix	2566	ERR111594	ERS075217	-	-
E_R29005	genital	E	Russia	F	2010	endocervix	2566	ERR111630	ERS082947	-	-
E_R30444	genital	E	Russia	F	2010	endocervix	2566	ERR111600	ERS075223	-	-
E_R32100	genital	E	Russia	F	2010	endocervix	2566	ERR111563	ERS075186	-	Seth-Smith 2013
E_R33420	genital	E	Russia	F	2010	endocervix	2566	ERR164655	ERS133242	-	-
E_R35067	genital	E	Russia	M	2010	urethra	2566	ERR111567	ERS075190	-	-
E_Rb1392	genital	E	UK	F	2010	endocervix	2566	ERR071994	ERS066957	-	-
E_Rb10387	genital	E	UK	M	2010	Unknown	2566	ERR034219	ERS017906	-	-
E_Rb10392	genital	E	UK	F	2010	endocervix	2566	ERR175633	ERS177819	-	-
E_S581	genital	E	Sweden	F	2011	endocervix	2566	ERR189737	ERS153014	-	-
E_S1019	genital	E	Sweden	F	2010	endocervix	2566	ERR140808	ERS095078	-	-
E_S1148	genital	E	Sweden	F	2010	endocervix	2566	ERR140810	ERS095080	-	-
E_S1227	genital	E	Sweden	F	2010	endocervix	2566	ERR140805	ERS095075	-	-
E_S1528	genital	E	Sweden	F	2010	endocervix	2566	ERR140809	ERS095079	-	-

E_S1613	genital	E	Sweden	F	2010	endocervix	2566	ERR140812	ERS095082	-	-
E_S1618	genital	E	Sweden	F	2010	endocervix	2566	ERR108290	ERS082972	-	-
E_S1886	genital	E	Sweden	F	2010	endocervix	2566	ERR108289	ERS082971	-	-
E_S2384	genital	E	Sweden	F	2010	endocervix	2566	ERR140760	ERS095030	-	-
E_S2491	genital	E	Sweden	F	2010	endocervix	2566	ERR108284	ERS082966	-	-
E_S2699	genital	E	Sweden	F	2011	endocervix	2566	ERR140831	ERS095101	-	-
E_S2713	genital	E	Sweden	F	2011	endocervix	2566	ERR140832	ERS095102	-	-
E_S3024	genital	E	Sweden	M	2010	conjunctiv:	2566	ERR111579	ERS075202	-	-
E_S3066	genital	E	Sweden	F	2011	endocervix	2566	ERR140841	ERS095111	-	-
E_S3073	genital	E	Sweden	F	2010	endocervix	2566	ERR140801	ERS095071	-	-
E_S3085	genital	E	Sweden	F	2011	endocervix	2566	ERR140833	ERS095103	-	-
E_S3122	genital	E	Sweden	F	2010	endocervix	2566	ERR111582	ERS075205	-	-
E_S3695	genital	E	Sweden	F	2006	endocervix	2566	ERR140815	ERS095085	-	-
E_S3711	genital	E	Sweden	F	2011	endocervix	2566	ERR140834	ERS095104	-	-
E_S3724	genital	E	Sweden	F	2006	endocervix	2566	ERR140816	ERS095086	-	-
E_S3732	genital	E	Sweden	F	2010	Unknown	2566	ERR108282	ERS082964	-	-
E_S4007	genital	E	Sweden	F	2006	endocervix	2566	ERR140817	ERS095087	-	-
E_S4106	genital	E	Sweden	F	2010	endocervix	2566	ERR140803	ERS095073	-	-
E_S4247	genital	E	Sweden	M	2010	urethra	2566	ERR108293	ERS082975	-	-
E_S4324	genital	E	Sweden	M	2010	urethra	2566	ERR108288	ERS082970	-	-
E_S4471	genital	E	Sweden	F	2010	endocervix	2566	ERR108285	ERS082967	-	-
E_SF4	genital	E	USA	F	2003	endocervix	2566	-	-	-	This study
E_SF9	genital	E	USA	F	2003	endocervix	2566	-	-	-	This study
E_SF13	genital	E	USA	F	2003	endocervix	2566	-	-	-	This study
E_SF14	genital	E	USA	F	2003	endocervix	2566	-	-	-	This study
E_SF15	genital	E	USA	F	2003	endocervix	2566	-	-	-	This study
E_SF17	genital	E	USA	F	2003	endocervix	2566	-	-	-	This study
E_SF18	genital	E	USA	F	2003	endocervix	2566	-	-	-	This study
E_SF23	genital	E	USA	F	2003	endocervix	2566	-	-	-	This study
E_SF24	genital	E	USA	F	2003	endocervix	2566	-	-	-	This study
E_SF26	genital	E	USA	F	2003	endocervix	2566	-	-	-	This study
E_SF28	genital	E	USA	F	2003	endocervix	2566	-	-	-	v
E_Soton17	genital	E	UK	F	2009	endocervix	2566	ERR111555	ERS075178	-	-
E_Soton53	genital	E	UK	F	2009	endocervix	2566	ERR111556	ERS075179	-	-
E_Soton83	genital	E	UK	F	2009	endocervix	2566	ERR140823	ERS095093	-	-
E_Soton107	genital	E	UK	F	2009	endocervix	2566	ERR111557	ERS075180	-	-
E_Soton116	genital	E	UK	F	2009	endocervix	2566	ERR111558	ERS075181	-	-
E_Soton120	genital	E	UK	F	2009	endocervix	2566	ERR111559	ERS075182	-	-
E_Soton145	genital	E	UK	F	2009	endocervix	2566	ERR111560	ERS075183	-	-
E_Soton159	genital	E	UK	F	2009	endocervix	2566	ERR111561	ERS075184	-	-
E_SotonE2	genital	E	UK	F	2009	endocervix	2566	ERR175612	ERS013789	-	-

E_SotonE4	genital	E	UK	F	2009	endocervix	2566	ERR026551	ERS013791	HE603232	Harris 2012
E_SotonE6	genital	E	UK	F	2009	endocervix	2566	ERR026543	ERS013793	-	-
E_SotonE8	genital	E	UK	F	2009	endocervix	2566	ERR027329	ERS008763	HE603233	Harris 2012
E_Sou60	genital	E	UK		1985	unknown	2566	ERR210999	ERS161049	-	-
E_Sou75	genital	E	UK		1985	unknown	2566	ERR211004	ERS161054	-	-
E_Sou102	genital	E	UK	F	1985	unknown	2566	ERR211010	ERS161060	-	-
E_STN2	genital	E	UK		1985	unknown	2566	ERR658574	ERS208554	-	-
E_STN10	genital	E	UK		1985	unknown	2566	ERR658575	ERS208555	-	-
E_STN11	genital	E	UK		1985	unknown	2566	ERR658576	ERS208556	-	-
E_STN12	genital	E	UK		1985	unknown	2566	ERR658577	ERS208557	-	-
E_STN47	genital	E	UK		1985	unknown	2566	ERR658581	ERS208562	-	-
E_STN68	genital	E	UK		1985	unknown	2566	ERR658582	ERS208563	-	-
E_STN92	genital	E	UK		1985	unknown	2566	ERR658583	ERS208564	-	-
E_STN115	genital	E	UK		1985	unknown	2566	ERR658587	ERS208568	-	-
E_STN119	genital	E	UK		1985	unknown	2566	ERR658588	ERS208569	-	-
E_SW2	genital	E	Sweden	M	2006	urethra	2566	ERR008596	ERS001397	FM865439	Unemo 2010
E_SW3	genital	E	Sweden	F	2001	endocervix	2566	ERR008589	ERS001406	FM865440	Harris 2012
E_Swab6	genital	E	UK	F	2010	vagina	2566	ERR175608	ERS177764	-	Seth-Smith 2013
E_SwabB4	genital	E	UK	F	2010	unknown	2566	ERR034345	ERS015770	-	Seth-Smith 2013
E_UK34334	genital	E	UK	M	2012	urethra	2566	ERR658682	ERS208409	-	-
E_UK466129	genital	E	UK	F	2012	Cx/Urethra	2566	ERR658591	ERS208318	-	-
E_UK466546	genital	E	UK	F	2012	Cx/Urethra	2566	ERR658639	ERS208366	-	-
E_UK582206	genital	E	UK	F	2012	Cx/Urethra	2566	ERR658642	ERS208369	-	-
E_UK582263	genital	E	UK	F	2012	Cx/Urethra	2566	ERR658595	ERS208322	-	-
E_UK584031	genital	E	UK	F	2012	Cx/Urethra	2566	ERR658602	ERS208329	-	-
E_UK663813	genital	E	UK	F	2012	Cx/Urethra	2566	ERR658606	ERS208333	-	-
E_UK663968	genital	E	UK	M	2012	urethra	2566	ERR658668	ERS208395	-	-
E_UK664394	genital	E	UK	M	2012	urethra	2566	ERR658669	ERS208396	-	-
E_UK769748	genital	E	UK	F	2012	Cx/Urethra	2566	ERR658670	ERS208397	-	-
E_UK769852	genital	E	UK	M	2012	urethra	2566	ERR658671	ERS208398	-	-
E_UK913723	genital	E	UK	F	2012	Cx/Urethra	2566	ERR658617	ERS208344	-	-
E_UK913953	genital	E	UK	F	2012	vagina	2566	ERR658619	ERS208346	-	-
F_70	genital	F				unknown	2566	-	-	ABYF01000001	Jeffrey 2010
F_1-93	genital	F	USA	F	1993	endocervix	2566	-	-	CP006671	Putman 2013
F_2-93	genital	F	USA	F	1993	endocervix	2566	-	-	CP006672	Putman 2013
F_6-94	genital	F	USA	F	1994	endocervix	2566	-	-	CP006673	Putman 2013
F_11-96	genital	F	USA	F	1996	endocervix	2566	-	-	CP006674	Putman 2013
F_6068	genital	F	Germany	F		urethra				CP015306	Eder 2017
F_AddT9	genital	F	UK			unknown	2566	ERR658365	ERS151259	-	-
F_Aus20	genital	F	Australia		1986-1989	unknown	2566	ERR189754	ERS153031	-	Andersson 2016
F_C55	genital	F	UK	F	2011	vagina	2566	ERR175625	ERS177811	-	-

F_CS84708	genital	F		F	2015	endocervix				CP010569	Borges 2015
F_Fin106	genital	F	Finland	F	2010	endocervix	2566	ERR278132	ERS200061	-	-
F_Fin152	genital	F	Finland	F	2010	endocervix	2566	ERR278139	ERS200068	-	-
F_Fin175	genital	F	Finland	F	2010	endocervix	2566	ERR278146	ERS200075	-	-
F_Fin181	genital	F	Finland	F	2010	endocervix	2566	ERR278162	ERS200091	-	-
F_Fin213	genital	F	Finland	F	2011	endocervix	2566	ERR278190	ERS200119	-	-
F_Fin219	genital	F	Finland	F	2011	endocervix	2566	ERR278160	ERS200089	-	-
F_IC-Cal-3	genital	F	USA		1960	ocular (nec	2566	ERR278181	ERS200110	-	Joseph 2012
F_It686	genital	F	Italy	F	2011	endocervix	2566	ERR658532	ERS208508	-	-
F_It688	genital	F	Italy	M	2011	urethra	2566	ERR140764	ERS095034	-	-
F_NI1	genital	F	Unknown			unknown	2566	ERR175650	ERS177836	-	-
F_NL30	genital	F	Netherlands	F	2001	endocervix	2566	ERR164690	ERS133277	-	-
F_NL31	genital	F	Netherlands	F	2001	endocervix	2566	ERR189762	ERS153039	-	-
F_NL35	genital	F	Netherlands	F	2001	endocervix	2566	ERR211022	ERS161072	-	-
F_NL36	genital	F	Netherlands	F	2001	endocervix	2566	ERR189726	ERS153003	-	-
F_NL38	genital	F	Netherlands	F	2001	endocervix	2566	ERR278184	ERS200113	-	-
F_R4663	genital	F	Russia	F	2011	endocervix	2566	ERR140795	ERS095065	-	-
F_R7369	genital	F	Russia	F	2011	endocervix	2566	ERR140798	ERS095068	-	-
F_R12921	genital	F	Russia	F	2011	endocervix	2566	ERR140799	ERS095069	-	-
F_R28312	genital	F	Russia	M	2010	urethra	2566	ERR111597	ERS075220	-	-
F_S1470	genital	F	Sweden	F	2010	endocervix	2566	ERR108292	ERS082974	-	-
F_S1494	genital	F	Sweden	F	2010	endocervix	2566	ERR140811	ERS095081	-	-
F_S2430	genital	F	Sweden	M	2010	urethra	2566	ERR111583	ERS075206	-	-
F_S2526	genital	F	Sweden	F	2010	unknown	2566	ERR111577	ERS075200	-	-
F_S2595	genital	F	Sweden	F	2010	endocervix	2566	ERR108283	ERS082965	-	
F_S3948	genital	F	Sweden	F	2010	endocervix	2566	ERR140800	ERS095070	-	-
F_S4410	genital	F	Sweden	F	2010	endocervix	2566	ERR111593	ERS075216	-	-
F_SF7	genital	F	USA	F	2003	endocervix	2566				Samboonna 2019
F_SF8	genital	F	USA	F	2003	endocervix	2566				Samboonna 2019
F_SF10	genital	F	USA	F	2003	endocervix	2574				Samboonna 2019
F_SF11	genital	F	USA	F	2003	endocervix	2580				Samboonna 2019
F_SF14	genital	F	USA	F	2003	endocervix	2574				Samboonna 2019
F_SF16	genital	F	USA	F	2003	endocervix	2566				Samboonna 2019
F_SF19	genital	F	USA	F	2003	endocervix	2566				Samboonna 2019
F_SF21	genital	F	USA	F	2003	endocervix	2574				Samboonna 2019
F_Soton18	genital	F	UK	F	2009	endocervix	2566	ERR026569	ERS013808	-	-
F_Soton48	genital	F	UK	F	2009	endocervix	2566	ERR140820	ERS095090	-	-
F_Soton88	genital	F	UK	F	2009	endocervix	2566	ERR026581	ERS013818	-	-
F_Soton106	genital	F	UK	F	2009	endocervix	2566	ERR140824	ERS095094	-	-
F_Soton118	genital	F	UK	F	2009	endocervix	2566	ERR140825	ERS095095	-	-
F_Soton137	genital	F	UK	F	2009	endocervix	2566	ERR140827	ERS095097	-	-

F_SotonF1	genital	F	UK	F	2009	endocervix	2566	ERR026556	ERS013796	-	-
F_SotonF2	genital	F	UK	F	2009	endocervix	2566	ERR026557	ERS013797	-	-
F_SotonF3	genital	F	UK	F	2009	endocervix	2566	ERR027330	ERS008764	HE603234	Harris 2012
F_SotonF4	genital	F	UK	F	2009	endocervix	2566	ERR026558	ERS013798	-	-
F_Sou9	genital	F	UK		1985	unknown	2566	ERR189771	ERS153048	-	-
F_Sou87	genital	F	UK		1985	unknown	2566	ERR211005	ERS161055	-	-
F_Sou89	genital	F	UK	F		unknown	2566	ERR211006	ERS161056	-	-
F_Sou100	genital	F	UK	M		unknown	2566	ERR211009	ERS161059	-	-
F_STN15	genital	F	UK		1985	unknown	2566	ERR658578	ERS208558	-	-
F_STN22	genital	F	UK		1985	unknown	2566	ERR658580	ERS208560	-	-
F_STN110	genital	F	UK		1985	unknown	2566	ERR658585	ERS208566	-	-
F_SW4	genital	F	Sweden	F	2002	endocervix	2566	ERR008588	ERS001414	FM865441	Harris 2012
F_SW5	genital	F	Sweden	F	2002	endocervix	2566	ERR008582	ERS001415	FM865442	Harris 2012
F_Swab5	genital	F	UK	F	2010	vagina	2566	ERR024700	ERS013115	-	Seth-Smith 2013
F_SwabB1	genital	F	UK	F	2010	vagina	2566	ERR173901	ERS177788	-	Seth-Smith 2013
F_SwabB8	genital	F	UK	F	2010	vagina	2566	ERR034346	ERS015772	-	Seth-Smith 2013
F_SWFP	genital	F	Sweden	F	2009	endocervix	2566	ERR008598	ERS001400	-	-
F_UK35155	genital	F	UK	F	2012	Cx/Urethra	2566	ERR658626	ERS208353	-	-
F_UK220521	genital	F	UK	M	2012	urethra	2566	ERR658399	ERS160285	-	-
F_UK465966	genital	F	UK	F	2012	Cx/Urethra	2566	ERR658632	ERS208359	-	-
F_UK466273	genital	F	UK	F	2012	Cx/Urethra	2566	ERR658592	ERS208319	-	-
F_UK583012	genital	F	UK	F	2012	Cx/Urethra	2566	ERR658656	ERS208383	-	-
F_UK583072	genital	F	UK	F	2012	Cx/Urethra	2566	ERR658652	ERS208379	-	-
F_UK583468	genital	F	UK	F	2012	Cx/Urethra	2566	ERR658649	ERS208376	-	-
F_UK584026	genital	F	UK	F	2012	Cx/Urethra	2566	ERR658647	ERS208374	-	-
F_UK663442	genital	F	UK	M	2012	urethra	2566	ERR658664	ERS208391	-	-
F_UK770010	genital	F	UK	F	2012	Cx/Urethra	2566	ERR658672	ERS208399	-	-
G_9301	genital	G	0	M		urethra	2566	-	-	CP001930	Jeffrey 2010
G_9768	genital	G	0	M		rectum	2566	-	-	CP001887	Jeffrey 2010
G_11074	genital	G	0	M		rectum	2566	-	-	CP001889	Jeffrey 2010
G_11222	genital	G	0	F		endocervix	2566	-	-	CP001888	Jeffrey 2010
G_Ar112	genital	G	Argentina	M	2007	urethra	2566	ERR111635	ERS082952	-	-
G_Ar246	genital	G	Argentina	M	2007	urethra	2566	ERR108276	ERS082958	-	-
G_Aus1	genital	G	Australia		1986-1989	unknown	2566	ERR189741	ERS153018	-	Andersson 2016
G_Aus16	genital	G	Australia		1986-1989	unknown	2566	ERR189751	ERS153028	-	Andersson 2016
G_Aus17	genital	G	Australia		1986-1989	unknown	2566	ERR189752	ERS153029	-	Andersson 2016
G_Aus18	genital	G	Australia		1986-1989	unknown	2566	ERR189759	ERS153036	-	Andersson 2016
G_Aus19	genital	G	Australia		1986-1989	unknown	2566	ERR189760	ERS153037	-	Andersson 2016
G_Fin144	genital	G	Finland	F	2010	endocervix	2566	ERR278137	ERS200066	-	-
G_Fin153	genital	G	Finland	F	2010	endocervix	2566	ERR278140	ERS200069	-	-
G_Fin158	genital	G	Finland	F	2010	endocervix	2566	ERR278142	ERS200071	-	-

G_Fin205	genital	G	Finland	F	2011	endocervix	2566	ERR278188	ERS200117	-	-
G_NL39	genital	G	Netherlands	F	2001	endocervix	2566	ERR189764	ERS153041	-	-
G_NL40	genital	G	Netherlands	F	2001	endocervix	2566	ERR189729	ERS153006	-	-
G_NL41	genital	G	Netherlands	F	2001	endocervix	2566	ERR210971	ERS161021	-	-
G_NL42	genital	G	Netherlands	F	2001	endocervix	2566	ERR210972	ERS161022	-	-
G_NL43	genital	G	Netherlands	F	2001	endocervix	2566	ERR189730	ERS153007	-	-
G_NL44	genital	G	Netherlands	F	2001	endocervix	2566	ERR278214	ERS200143	-	-
G_NL45	genital	G	Netherlands	F	2001	endocervix	2566	ERR189732	ERS153009	-	-
G_NL46	genital	G	Netherlands	F	2001	endocervix	2566	ERR189733	ERS153010	-	-
G_NL47	genital	G	Netherlands	F	2001	endocervix	2566	ERR210973	ERS161023	-	-
G_NL48	genital	G	Netherlands	F	2001	endocervix	2566	ERR189734	ERS153011	-	-
G_R297	genital	G	Russia	F	2011	endocervix	2566	ERR111576	ERS075199	-	-
G_R459	genital	G	Russia	F	2011	endocervix	2566	ERR111617	ERS082934	-	-
G_R2247	genital	G	Russia	F	2011	endocervix	2566	ERR111620	ERS082937	-	-
G_R3059	genital	G	Russia	F	2011	endocervix	2566	ERR111612	ERS082929	-	Seth-Smith 2013
G_R4175	genital	G	Russia	F	2011	endocervix	2566	ERR111614	ERS082931	-	Seth-Smith 2013
G_R9069	genital	G	Russia	F	2011	endocervix	2566	ERR111625	ERS082942	-	-
G_R9892	genital	G	Russia	M	2011	urethra	2566	ERR111619	ERS082936	-	-
G_R15108	genital	G	Russia	M	2011	urethra	2566	ERR140756	ERS095026	-	-
G_R23736	genital	G	Russia	F	2010	endocervix	2566	ERR111595	ERS075218	-	-
G_R27757	genital	G	Russia	F	2010	endocervix	2566	ERR111623	ERS082940	-	-
G_R30591	genital	G	Russia	F	2010	endocervix	2566	ERR111569	ERS075192	-	-
G_R31458	genital	G	Russia	M	2010	urethra	2566	ERR111605	ERS082922	-	-
G_R35506	genital	G	Russia	F	2010	endocervix	2566	ERR111610	ERS082927	-	-
G_R36176	genital	G	Russia	F	2010	endocervix	2566	ERR111611	ERS082928	-	Seth-Smith 2013
G_S1471	genital	G	Sweden	F	2010	endocervix	2566	ERR108291	ERS082973	-	-
G_S1824	genital	G	Sweden	F	2010	endocervix	2566	ERR111587	ERS075210	-	-
G_S1846	genital	G	Sweden	F	2010	endocervix	2566	ERR140807	ERS095077	-	-
G_S2477	genital	G	Sweden	F	2010	endocervix	2566	ERR140814	ERS095084	-	-
G_S2956	genital	G	Sweden	F	2010	conjunctiv	2566	ERR111581	ERS075204	-	-
G_S3270	genital	G	Sweden	F	2010	endocervix	2566	ERR111588	ERS075211	-	-
G_S3344	genital	G	Sweden	F	2010	endocervix	2566	ERR111591	ERS075214	-	-
G_S4641	genital	G	Sweden	F	2010	endocervix	2566	ERR140813	ERS095083	-	-
G_S4658	genital	G	Sweden	M	2010	conjunctiv	2566	ERR108295	ERS082977	-	-
G_SF20	genital	G	USA	F	2003	endocervix	2566				This study
G_SF21	genital	G	USA	F	2003	endocervix	2566				This study
G_Soton144	genital	G	UK	F	2009	endocervix	2566	ERR140828	ERS095098	-	-
G_SotonG1	genital	G	UK	F	2009	endocervix	2566	ERR026560	ERS013800	-	Harris 2012
G_UK22140	genital	G	UK			unknown		ERR658523	ERS160283		
G_UK58250	genital	G	UK	M	2012	urethra	2566	ERR658658	ERS208385	-	-
G_UK75036	genital	G	UK	F	2012	endocervix	2566	ERR658369	ERS160255	-	-

G_UK913362	genital	G	UK	F	2012	Cx/Urethra	2566	ERR658677	ERS208404	-	-
G_UW57	genital	G	USA		1971	endocervix	2566	ERR164658	ERS133245	-	Joseph 2012
H_Fin109	genital	H	Finland	F	2010	endocervix	2566	ERR278213	ERS200142	-	-
H_NL49	genital	H	Netherlands	F	2001	endocervix	2566	ERR210974	ERS161024	-	-
H_NL50	genital	H	Netherlands	F	2001	endocervix	2566	ERR210975	ERS161025	-	-
H_NL51	genital	H	Netherlands	F	2001	endocervix	2566	ERR210976	ERS161026	-	-
H_NL53	genital	H	Netherlands	F	2001	endocervix	2566	ERR210978	ERS161028	-	-
H_NL54	genital	H	Netherlands	F	2001	endocervix	2566	ERR210979	ERS161029	-	-
H_NL56	genital	H	Netherlands	F	2001	endocervix	2566	ERR210981	ERS161031	-	-
H_R13670	genital	H	Russia	M	2011	urethra	2566	ERR164654	ERS133241	-	-
H_R25308	genital	H	Russia	F	2010	endocervix	2566	ERR111616	ERS082933	-	-
H_R27887	genital	H	Russia	F	2010	endocervix	2566	ERR111599	ERS075222	-	-
H_R31975	genital	H	Russia	F	2010	endocervix	2566	ERR111606	ERS082923	-	-
H_S269	genital	H	Sweden	F	2011	unknown	2566	ERR164646	ERS133233	-	-
H_S1026	genital	H	Sweden	F	2010	endocervix	2566	ERR108301	ERS082983	-	-
H_S1314	genital	H	Sweden	F	2010	endocervix	2566	ERR108296	ERS082978	-	-
H_S1432	genital	H	Sweden	F	2010	endocervix	2566	ERR108303	ERS082985	-	-
H_S4377	genital	H	Sweden	F	2010	endocervix	2566	ERR164648	ERS133235	-	-
H_UW4	genital	H	USA	F	1965	endocervix	2566	ERR558497	ERS177776	-	Joseph 2012
H_UW4	genital	H	USA	F	1965	endocervix	2566	ERR558497	ERS177776	-	Joseph 2012
Ia_20-97	genital	I-Ia	USA			endocervix	2566	-	-	CP006678	Putman 2013
Ia/CS190/96		I-Ia		F	2015	endocervix				CP010571	Borges 2015
Ia_SF16	genital	Ia	USA	F	2003	endocervix	2566				
Ia_SF25	genital	Ia	USA	F	2003	endocervix	2566				
Ia_SF27	genital	Ia	USA	F	2003	endocervix	2566				
Ia_SotonIa1	genital	I-Ia	UK	F	2009	endocervix	2566	ERR026555	ERS013804	HE603236	Harris 2012
Ia_SotonIa3	genital	I-Ia	UK	F	2009	endocervix	2566	ERR026565	ERS013805	HE603237	Harris 2012
Ia_UW202	genital	I-Ia	USA			unknown					This study
I_NL58	genital	I-Ia	Netherlands	F	2001	endocervix	2566	ERR210982	ERS161032	-	-
I_NL63	genital	I-Ia	Netherlands	F	2001	endocervix	2566	ERR210985	ERS161035	-	-
I_NL66	genital	I-Ia	Netherlands	F	2001	endocervix	2566	ERR211026	ERS161076	-	-
I_NL67	genital	I-Ia	Netherlands	F	2001	endocervix	2566	ERR210986	ERS161036	-	-
I_NL69	genital	I-Ia	Netherlands	F	2001	endocervix	2566	ERR211023	ERS161073	-	-
I_NL70	genital	I-Ia	Netherlands	F	2001	endocervix	2566	ERR210987	ERS161037	-	-
I_NL72	genital	I-Ia	Netherlands	F	2001	endocervix	2566	ERR211027	ERS161077	-	-
I_S2459	genital	I-Ia	Sweden	F	2010	endocervix	2566	ERR111592	ERS075215	-	-
I_UK913341	genital	I-Ia	UK	F	2012	vagina	2566	ERR658613	ERS208340		
I_UW12	genital	I-Ia	USA		1966	urethra	2566	ERR278215	ERS200144	-	-
Ja/UW-92	genital	Ja	Seattle, USA		1992	unknown	2566				This study
J_27-97	genital	J	USA			endocervix	2566	-	-	CP006679	Putman 2013
J_31-98	genital	J	USA			endocervix	2566	-	-	CP006680	Putman 2013

J_6276	genital	J	0	F		endocervix	2566	-	-	ABYD01000001	Jeffrey 2010
J 6276s	genital					endocervix	2566			ABYD01000002	Jeffrey 2010
J_C114	genital	J	UK	F	2011	vagina	2566	ERR175629	ERS177815	-	-
J_NL55	genital	J	Netherlands	F	2001	endocervix	2566	ERR210980	ERS161030	-	-
J_NL76	genital	J	Netherlands	F	2001	endocervix	2566	ERR210989	ERS161039	-	-
J_NL78	genital	J	Netherlands	F	2001	endocervix	2566	ERR210990	ERS161040	-	-
J_S42	genital	J	Sweden	F	2011	endocervix	2566	ERR140818	ERS095088	-	-
J_S178	genital	J	Sweden	F	2011	endocervix	2566	ERR140829	ERS095099	-	-
J_S1254	genital	J	Sweden	F	2010	endocervix	2566	ERR108302	ERS082984	-	-
J_S3107	genital	J	Sweden	F	2010	endocervix	2566	ERR108299	ERS082981	-	-
J_S4281	genital	J	Sweden	F	2010	endocervix	2566	ERR108300	ERS082982	-	-
J_S4821	genital	J	Sweden	F	2010	endocervix	2566	ERR164649	ERS133236	-	-
J_SF5	genital	J	USA	F	2003	endocervix	2566				This study
J_SF6	genital	J	USA	F	2003	endocervix	2566				This study
J_Sou106	genital	J	UK	F	1985	unknown	2566	ERR211013	ERS161063	-	-
J_UK583676	genital	J	UK	M	2012	urethra	2566	ERR658657	ERS208384	-	-
J_UK913454	genital	J	UK	M	2012	urethra	2566	ERR658679	ERS208406	-	-
J_UW36	genital	J	USA	F	1971	endocervix	2566	ERR558504	ERS177783	-	-
K_Ar74	genital	K	Argentina	F	2005	ocular	2566	ERR658565	ERS208545		
K_Ar650	genital	K	Argentina	M	2006	urethra	2566	ERR658566	ERS208546	-	-
K_Fin128	genital	K	Finland	F	2010	endocervix	2566	ERR278186	ERS200115	-	-
K_Fin139	genital	K	Finland	F	2010	endocervix	2566	ERR278163	ERS200092	-	-
K_Fin202	genital	K	Finland	F	2011	endocervix	2566	ERR278164	ERS200093	-	-
K_Fin204	genital	K	Finland	F	2011	endocervix	2566	ERR278165	ERS200094	-	-
K_NL81	genital	K	Netherlands	F	2001	endocervix	2566	ERR211028	ERS161078	-	-
K_NL82	genital	K	Netherlands	F	2001	endocervix	2566	ERR211024	ERS161074	-	-
K_NL83	genital	K	Netherlands	F	2001	endocervix	2566	ERR211029	ERS161079	-	-
K_NL84	genital	K	Netherlands	F	2001	endocervix	2566	ERR211025	ERS161075	-	-
K_NL85	genital	K	Netherlands	F	2001	endocervix	2566	ERR210991	ERS161041	-	-
K_NL87	genital	K	Netherlands	F	2001	endocervix	2566	ERR211030	ERS161080	-	-
K_R2084	genital	K	Russia	F	2011	endocervix	2566	ERR111622	ERS082939	-	-
K_R11642	genital	K	Russia	F	2011	endocervix	2566	ERR140839	ERS095109	-	-
K_R13207	genital	K	Russia	F	2011	endocervix	2566	ERR140836	ERS095106	-	-
K_R14876	genital	K	Russia	M	2011	urethra	2566	ERR140838	ERS095108	-	-
K_R15212	genital	K	Russia	F	2011	endocervix	2566	ERR140840	ERS095110	-	-
K_R26881	genital	K	Russia	F	2010	endocervix	2566	ERR111626	ERS082943	-	-
K_R27128	genital	K	Russia	F	2010	endocervix	2563	ERR111629	ERS082946	-	-
K_R32840	genital	K	Russia	F	2010	endocervix	2566	ERR111627	ERS082944	-	-
K_R34345	genital	K	Russia	F	2010	endocervix	2566	ERR111609	ERS082926	-	-
K_R34962	genital	K	Russia	M	2010	urethra	2566	ERR111608	ERS082925	-	-
K_R35248	genital	K	Russia	M	2010	urethra	2566	ERR111628	ERS082945	-	-

K_S143	genital	K	Sweden	F	2011	endocervix	2566	ERR108304	ERS082986	-	-
K_S4034	genital	K	Sweden	F	2010	endocervix	2566	ERR164647	ERS133234	-	-
K_S4229	genital	K	Sweden	F	2010	urethra	2566	ERR111584	ERS075207	-	-
K_SotonK1	genital	K	UK	F	2009	endocervix	2566	ERR026559	ERS013799	HE603238	Harris 2012
K_UK58323	genital	K	UK	F	2012	Cx/Urethra	2566	ERR658600	ERS208327	-	-
K_UK66306	genital	K	UK	F	2012	Cx/Urethra	2566	ERR658662	ERS208389	-	-
K_UK66312	genital	K	UK	F	2012	Cx/Urethra	2566	ERR658660	ERS208387	-	-
K_UK76907	genital	K	UK	F	2012	Urine	2566	ERR658561	ERS208541	-	-
K_UW31	genital	K	USA	F	1973	endocervix	2566	ERR558505	ERS177784	-	-
L1_L82	LGV	L1	South Africa	M	1985	urethra	2566	ERR211057	ERS161107	-	-
L1_L115p10	LGV	L1	South Africa	M	1986	urethra	2566	ERR211062	ERS161112	-	-
L1_L146	LGV	L1	South Africa	M	1986	ulcer	2566	ERR211044	ERS161094	-	-
L1_L165	LGV	L1	South Africa	M	1986	ulcer	2566	ERR211045	ERS161095	-	-
L1_L224	LGV	L1	South Africa	M	1986	urethra	2566	ERR211058	ERS161108	-	-
L1_L232	LGV	L1	South Africa	M	1987	urethra	2566	ERR211059	ERS161109	-	-
L1_L246	LGV	L1	South Africa	M	1987	urethra	2566	ERR211046	ERS161096	-	-
L1_L867	LGV	L1	South Africa	M	1993	urethra	2566	ERR211060	ERS161110	-	-
L1_L942	LGV	L1	South Africa	M	1994	urethra	2566	ERR211061	ERS161111	-	-
L1_L1034	LGV	L1	South Africa	M	1994	urethra	2566	ERR211035	ERS161085	-	-
L1_LGV98	LGV	L1	South Africa	M		unknown	2566	ERR071990	ERS066953	-	Seth-Smith 2013
L1_LGV913	LGV	L1	South Africa	M		unknown	2566	ERR071991	ERS066954	-	Seth-Smith 2013
L1_SA160	LGV	L1	South Africa	M	1986	ulcer	2566	ERR658551	ERS208531	-	-
L1_SA409	LGV	L1	South Africa	M	1990	ulcer	2566	ERR658553	ERS208533	-	-
L1_SABY21	LGV	L1	South Africa	M	1999	ulcer	2566	ERR658549	ERS208529	-	-
L1_115	LGV	L1	South Africa			unknown	2566	ERR008593	ERS001411	HE603218	Harris 2012
L1_224	LGV	L1	South Africa			unknown	2566	ERR008580	ERS001404	HE603220	Harris 2012
L1_440	LGV	L1	USA	M	1968	lymph nod	2566	ERR008595	ERS001396		Harris 2012
L1_1333p2	LGV	L2	South Africa	M		genital ulc	2566			HE601951	
L1_Ur58380	LGV	L1	UK	M	2012	Urine	2566	ERR658557	ERS208537	HE603228	Harris 2012
L2b_795	LGV	L2b	France	M	2004	rectum	2566	ERR008586	ERS001409		Harris 2012
L2b_8200	LGV	L2b	Sweden	M	2007	proctitis	2566	ERR021952	ERS004108		Harris 2012
L2b_8200-07	LGV	L2b	Sweden	M	2007	proctitis	2566	ERR021952	ERS004108		Harris 2012
L2b_Ams1	LGV	L2b	Netherlands	M	2004	penile ulce	2566			HE601959	Harris 2012
L2b_Ams2	LGV	L2b	Netherlands	M	2005	anus	2566			HE601961	Harris 2012
L2b_Ams3	LGV	L2b	Netherlands	M	2004	anus	2566			HE601962	Harris 2012
L2b_Ams4	LGV	L2b	Netherlands	M	2005	anus	2566			HE601964	Harris 2012
L2b_Ams5	LGV	L2b	Netherlands	M	2004	anus	2566			HE601965	Harris 2012
L2b_C1	LGV	L2b	Canada	M	2004	rectum	2566	ERR008579	ERS001398		Harris 2012
L2b_C2	LGV	L2b	Canada	M	2005	rectum	2566	ERR008592	ERS001399		Harris 2012
L2b_Canada1	LGV	L2b	Canada	M	2004	rectum	2566			HE601963	Harris 2012
L2b_Canada2	LGV	L2b	Canada	M	2005	rectum	2566			HE601957	Harris 2012

L2b_CC37	LGV	L2b	UK	M	2011	rectum	2566	ERR140847	ERS095117		Harris 2012
L2b_CS1908	LGV	L2b	Portugal	M	2015	anorectal	2566			CP009923	Borges 2015
L2b_CS7840	LGV	L2b	Portugal		2015	proctitis	2566			CP009925	Borges 2016
L2b_CV204	LGV	L2b	France	M	2006	rectum	2566	ERR019531	ERS003307		Harris 2012
L2b_H17IMS	LGV	L2b	UK	M	2008	rectum	2566	ERR140766	ERS095036		
L2b_HPA1	LGV	L2b	UK	M	2005	rectum	2566	ERR164659	ERS133246		
L2b_HPA21	LGV	L2b	UK	M	2009	rectum	2566	ERR140767	ERS095037		
L2b_HPA27	LGV	L2b	UK	M	2005	rectum	2566	ERR164661	ERS133248		
L2b_HPA29	LGV	L2b	UK	M	2004	rectum	2566	ERR164662	ERS133249		
L2b_HPA31	LGV	L2b	UK	M	2005	rectum	2566	ERR164663	ERS133250		Thomson 2008
L2b_HPA34	LGV	L2b	UK	M	2008	rectum	2566	ERR164665	ERS133252		Harris 2012
L2b_LST	LGV	L2b	France	M	2008	rectum	2566	ERR019528	ERS003315		Somboonna 2011
L2b_s11	LGV	L2b	Netherlands	M	2004	penile ulce	2566	ERR008590	ERS001408	HE603228	Harris 2012
L2b_s121	LGV	L2b	Netherlands	M	2005	anal swab	2566	ERR008597	ERS001402		
L2b_s300	LGV	L2b	Netherlands	M	2004	anal swab	2566	ERR008599	ERS001413		
L2b_s750	LGV	L2b	Netherlands	M	2004	anal swab	2566	ERR008594	ERS001410		
L2b_s906	LGV	L2b	Netherlands	M	2005	anal swab	2566	ERR008584	ERS001395		
L2b_SF4180	LGV	L2b	USA	M	1984	rectum	2566	ERR348840	ERS248048		
L2b_SF4644	LGV	L2b	USA	M	1985	rectum	2566	ERR348841	ERS248049		
L2b_SF1565	LGV	L2b	USA	M	2001	rectum	2566	ERR516390	ERS373084		
L2b_SF1567	LGV	L2b	USA	M	2003	rectum	2566	ERR516391	ERS373085		
L2b_SF1567	LGV	L2b	USA	M	2003	rectum	2566	ERR348858	ERS248080		
L2b_UCH1	LGV	L2b	UK	M	2006	proctitis	2566	ERR008581	ERS001407		
L2b_UCH2	LGV	L2b	UK			proctitis	2566	ERR008587	ERS001405		
L2c	LGV	L2	USA	M	2000s	rectum	2566			CP002024	Somboonna 2011
L2_L198	LGV	L2	South Africa	M	1986	ulcer	2566	ERR211050	ERS161100		Seth-Smith 2013
L2_L694	LGV	L2	South Africa	M	1993	urethra	2566	ERR211064	ERS161114		Seth-Smith 2014
L2_LGV173	LGV	L2	South Africa	M		NA	2566	ERR071989	ERS066952		Seth-Smith 2015
L2_SF25667	LGV	L2	USA	M	1981	rectum	2566	ERR351533	ERS248067		Seth-Smith 2016
L2_SF40369	LGV	L2	USA	M	1984	rectum	2566	ERR348839	ERS248047		Seth-Smith 2017
L2_SFUW39	LGV	L2	USA	M	1980s	rectum	2566	ERR348842	ERS248050		Seth-Smith 2018
L2_434Bu	LGV	L2	USA	M	1968	bubo	2566			AM884176	Harris 2012
L2_434Bu(f)	LGV	L2	USA	M	1968	inguinal bu	2566			CP003963	
L2_434Bu(i)	LGV	L2	USA	M	1968	inguinal bu	2566			CP003965	
L2_470LN87	LGV	L2	USA	M	1968	lymph nod	2566	ERR348847	ERS248055		Harris 2012
L2_514BU11	LGV	L2	USA	M	1968	bubo	2566	ERR348844	ERS248052		
L2_526BU5	LGV	L2	USA	M	1968	bubo	2566	ERR516392	ERS373086		Harris 2012
L3_404	LGV	L3	USA	M	1967	lymph nod	2576	ERR008583	ERS001416	HE601955	
