# Supplementary material for: Tryptophan Operon Diversity Reveals Evolutionary Trends among Geographically Disparate Chlamydia trachomatis Ocular and Urogenital Strains Affecting Tryptophan Repressor and Synthase Function
Source: mBio. 2021 May 11;12(3):e00605-21. doi: 10.1128/mBio.00605-21 (PMC8262981; doi:10.1128/mBio.00605-21)
Supplement: TABLE S2 [file mbio.00605-21-st002.pdf]

Table S2. Secondary analysis of putative *C. trachomatis* recombinants involving the tryptophan operon identified by RDP4 using Recco.

**Sequence name**	**Start**[*]	**End**[*]	**Savings**[^]	**Aln pv**[#]	**Seq pv**[~]	**overall p-value**	**Mutation Cost**
B_QH111L	1935	2291	2	1	0.23077	1	12
B_QH111L	107	245	1	1	0.93507		11
I_UK913341	246	314	2	1	0.2997	1	10
I_UK913341	332	531	2	1	0.2997		10
I_UK913341	2472	2473	0.4	1	1		9.2

*Range of nucleotide positions where a recombination breakpoint could occur.
^Amount of mutation cost saved by introducing this recombination.
#p-value of this recombination event regarding savings distribution over the entire alignment - a very conservative measure for recombination.
~p-value of this recombination event regarding savings distribution over recombinant.
