# Supplementary material for: Tryptophan Operon Diversity Reveals Evolutionary Trends among Geographically Disparate Chlamydia trachomatis Ocular and Urogenital Strains Affecting Tryptophan Repressor and Synthase Function
Source: mBio. 2021 May 11;12(3):e00605-21. doi: 10.1128/mBio.00605-21 (PMC8262981; doi:10.1128/mBio.00605-21)
Supplement: TABLE S3 [file mbio.00605-21-st003.pdf]

Table S3. Nucleotide polymorphisms in *trpB* for *C. trachomatis* clinical and reference ocular strains.

Lineage	Site of infection	Unique *trpB* strain variants	Year of isolation	Geography	Number of variants	Variant ID	Year of isolation	Geography	Changes to *trpB*	Length of *trpB* (bp)
Ocular	Ocular	**A_HAR13**	1958	Egypt	7	A_D213, A_D230, A_SA1,	1957, 2001	Gambia, Saudi A	None	1179
Ocular	Ocular	A_2497	2000	Tanzania	47	A_363, A_5291, A_7249, A_MH858, A_MH1364, A_MH2145, A_MH3234, A_MH2497, A_MH4510, A_MH5368, A_MH5786, A_MH6446, A_MH7205, A_MH8910, A_MH9922, A_MH10549, A_MH10648, A_MH10901, A_MH11715, A_MH11979, A_MH12023, A_MH13849, A_MH14553, A_MH15048, A_MH15741, A_MH16005, A_MH16170, A_MH16665, A_MH17127_A_MH18843, A_MH18876, A_MH19676, A_MH20130, A_MH20933, A_MH21571, A_MH23527, A_MH24519, A_MH24640, A_MH24673, A_MH25256, A_MH25883, A_MH27137, A_MH35739, A_MH47300, A_MH53658, A_MH19657, A_MH26862	2000	Tanzania	931G>A; Nonsynonymous substitution	1179
Ocular	Ocular	A_SB002739	2013	Solomon Island	4	A_SB006930, A_SB008107, A_SB13112, A_SB13321	2013	Solomon Island	1141C>T; Nonsynonymous substitution	1179
Ocular	Ocular	**B_HAR36***	1969	Saudi Arabia	1	B_Jali16	1985	Gambia	None	1179
Ocular	Ocular	B_TZ1A828	1998	Tanzania	None	None	None	None	8-28del; frameshift and early truncation	33
Urogenital	Ocular	B_QH111L	2016	China	None	None	None	None	58C>T, 137A>G; Synonymous and nonsynonymous substitution	603
Ocular	Ocular	B_M48	2007	Gambia	None	None	None	None	557-558insGA; early truncation	570
Ocular	Ocular	B_Jali20	1985	Gambia	None	None	None	None	986G>C	1179
Ocular	Ocular	**Ba_Apache2**	1960	USA	None	None	None	None	1141C>T; Nonsynonymous substitution	1179
Ocular	Ocular	**C_TW3**	1959	Taiwan	None	None	None	None	None	1179
Ocular	Ocular	C_UW10	1964	Canada	None	None	None	None	179G>A; synonymous substitution	1179
Ocular	Ocular	**Da_TW448**	1985	Taiwan	None	None	None	None	None	1179

Note: trpB truncations are seen in B_TZ1A828, B_QH111L, B_M48.

*Since *Ct* reference strain B_UW5OT lacks trpB gene, B_HAR-36 was used as the reference strain for B genotype strains.
