# Supplementary material for: Tryptophan Operon Diversity Reveals Evolutionary Trends among Geographically Disparate Chlamydia trachomatis Ocular and Urogenital Strains Affecting Tryptophan Repressor and Synthase Function
Source: mBio. 2021 May 11;12(3):e00605-21. doi: 10.1128/mBio.00605-21 (PMC8262981; doi:10.1128/mBio.00605-21)
Supplement: FIG S1 [file mbio.00605-21-sf001.pdf]

Supplementary Figures

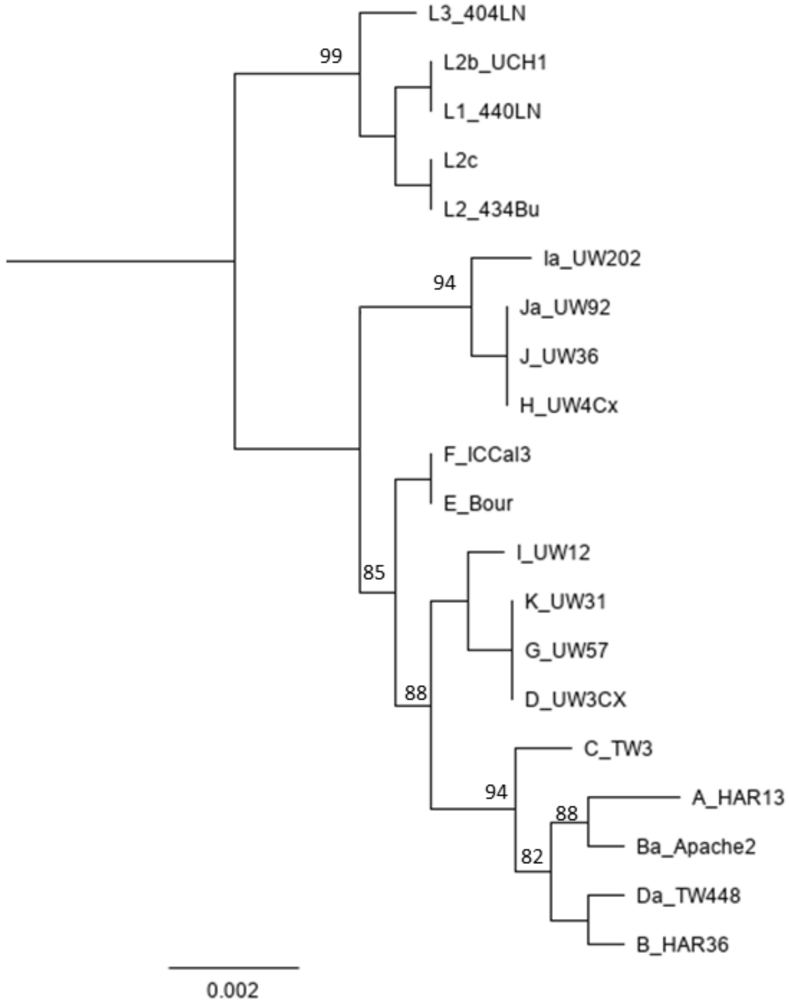

Supplementary Fig 1. Concatenated *trp*RBA phylogenetic tree of 21 *Ct* reference strains constructed by FastTree with a Generalized Time-Reversible model based on a MAFFT alignment of *trp*RBA sequences (see Methods). Both were executed in Geneious (https://www.geneious.com). The tree scale indicates the distance between the sequences and the branch length indicates the number of substitutions that have occurred in that branch.
