# Supplementary material for: Tryptophan Operon Diversity Reveals Evolutionary Trends among Geographically Disparate Chlamydia trachomatis Ocular and Urogenital Strains Affecting Tryptophan Repressor and Synthase Function
Source: mBio. 2021 May 11;12(3):e00605-21. doi: 10.1128/mBio.00605-21 (PMC8262981; doi:10.1128/mBio.00605-21)
Supplement: FIG S2 [file mbio.00605-21-sf002.pdf]

Chain A

mAQQSPYSAAMAEQRHQEWLRFVDLLKNAYQNDLHLPLLNLMLLPDEREALGTRVRIVEELLRGEMSQRELKNELGAGIATITRGSNYLKAAPVELRQWLEEVLLksdlehhhhhh

IAC2 R IV T RG Y

IAC1 LMLL

DBS1 QRELKNELGAGIATITRGSNYLKA

EDO PVE R

Chain B

mAQQSPYSAAMAEQRHQEWLRFVDLLKNAYQNDLHLPLLNLMLLPDEREALGTRVRIVEELLRGEMSQRELKNELGAGIATITRGSNYLKAAPVELRQWLEEVLLksdlehhhhhh

IAC1 R IV T RG Y

IAC2 LMLL

DBS2 QRELKNELGAGIATITRGSNYLKA

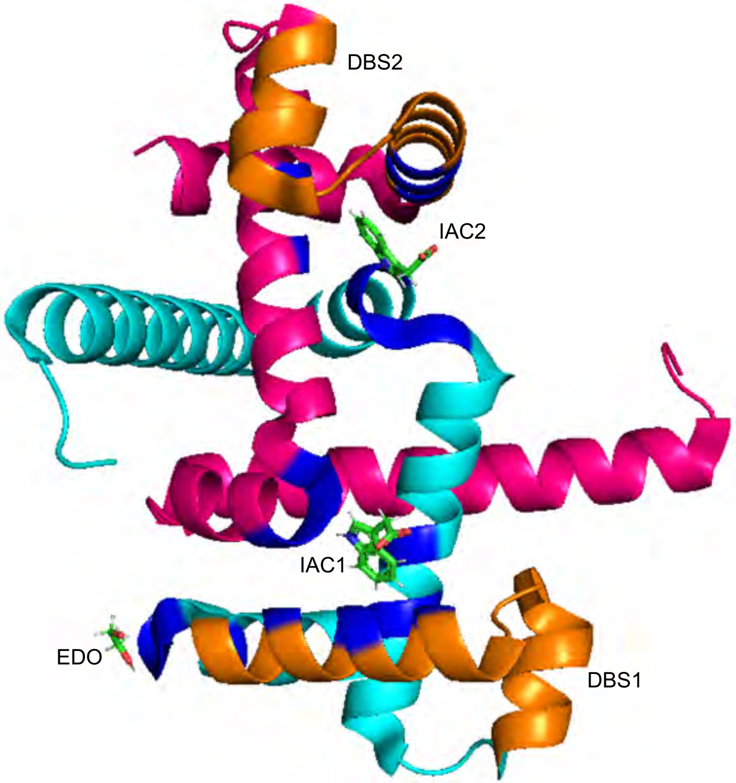

Supplementary Fig 2. Crystal structure of tryptophan repressor in *E.coli*, PDB: 6ENI. The two monomer subunits of the symmetric dimer are shown as cartoon diagrams, chain A in cyan and chain B in pink. Two molecules of indol-3-acetic acid (IAC) is bound at chain A and chain B, the 1,2-ethanediol (EDO) is bound at chain A only. The DNA binding motif (DBS) involved in the protein-DNA (operator binding) interactions is shown in orange and is present in chain A and B. The putative ligand binding sites for IAC and EDO are shown in blue. The reference sequence of *E.coli* TrpR 6eni with the LBS (blue), DBS (orange) and residues involved in dimerization (bold) are shown as well. Each molecule of the ligand IAC has LBS on chain A and chain B, respectively.
