# Supplementary material for: Tryptophan Operon Diversity Reveals Evolutionary Trends among Geographically Disparate Chlamydia trachomatis Ocular and Urogenital Strains Affecting Tryptophan Repressor and Synthase Function
Source: mBio. 2021 May 11;12(3):e00605-21. doi: 10.1128/mBio.00605-21 (PMC8262981; doi:10.1128/mBio.00605-21)
Supplement: FIG S3 [file mbio.00605-21-sf003.pdf]

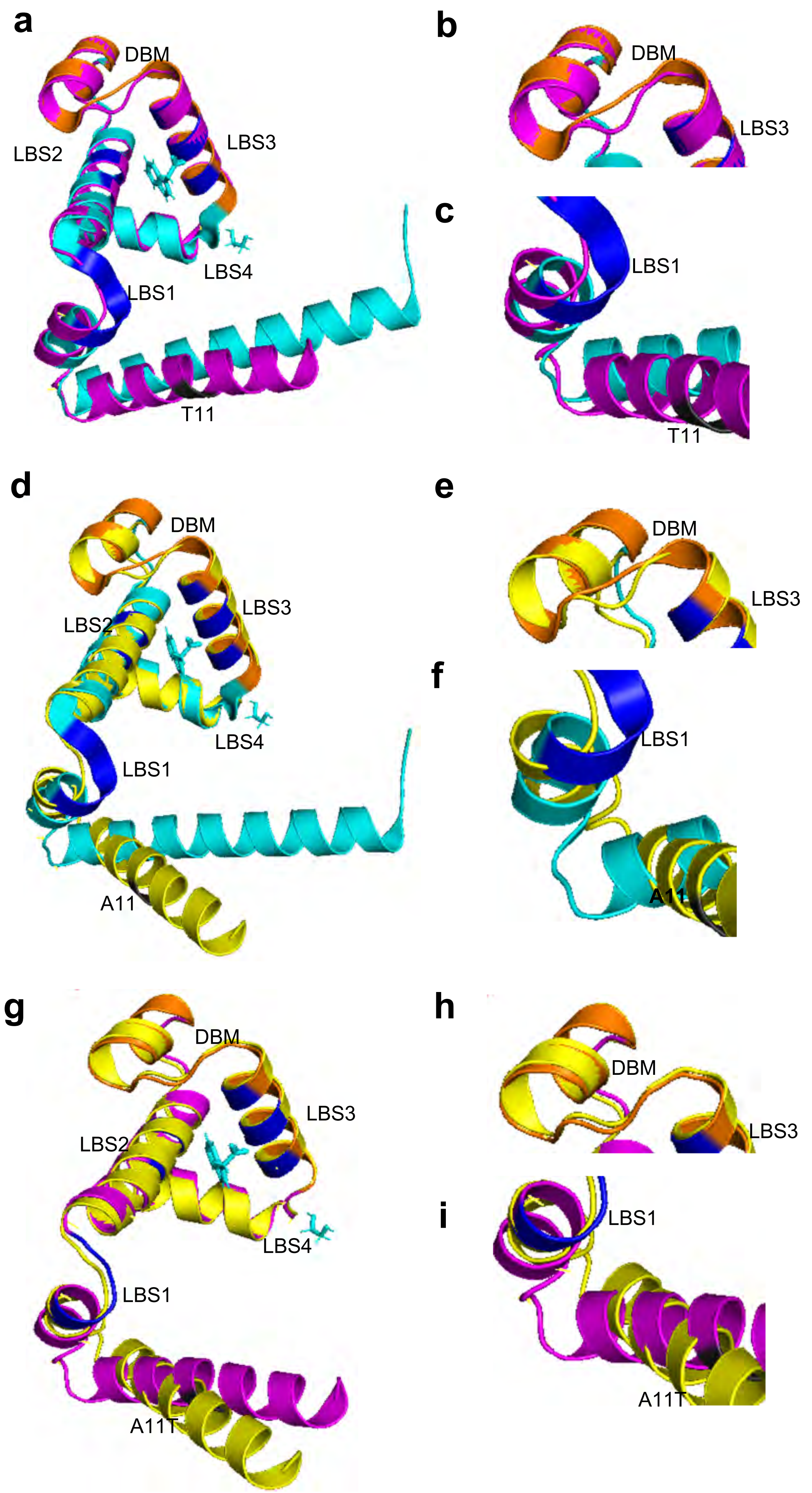
a
DBM
LBS2
LBS3
LBS4
LBS1
T11
b
DBM
LBS3
c
LBS1
T11
d
DBM
LBS3
LBS2
LBS4
LBS1
A11
e
DBM
LBS3
f
LBS1
A11
g
DBM
LBS3
LBS2
LBS4
LBS1
A11T
h
DBM
LBS3
i
LBS1
A11T

Supplementary Fig 3. TrpR 3D predicted structures of *Ct* clinical F_SwabB8 and reference F_IC-Cal-3 strains (A-I). (A) TrpR structure of *Ct* mutant strain F_SwabB8 (magenta) superimposed on the template 6eniA (cyan), with DBM in orange. The LBS 1, 2, 3 and 4 are shown in dark blue, and the aa substitution in relation to F_IC-Cal-3 at T11 is in black. (B and C) Structural changes in DBM and LBS1, respectively, of the mutant are shown. (D) TrpR structure of F_IC-Cal-3 (yellow) superimposed on the template 6eniA (cyan) with annotations as per A, B and C. (E and F) Structural changes in DBM and LBS1 of E_Bour. (G) TrpR 3D predicted structures of F_SwabB8 superimposed on F_IC-Cal-3 with structural changes noted in relation to (H) DBM based and (I) LBS1.
