# Supplementary material for: Tryptophan Operon Diversity Reveals Evolutionary Trends among Geographically Disparate Chlamydia trachomatis Ocular and Urogenital Strains Affecting Tryptophan Repressor and Synthase Function
Source: mBio. 2021 May 11;12(3):e00605-21. doi: 10.1128/mBio.00605-21 (PMC8262981; doi:10.1128/mBio.00605-21)
Supplement: FIG S4 [file mbio.00605-21-sf004.pdf]

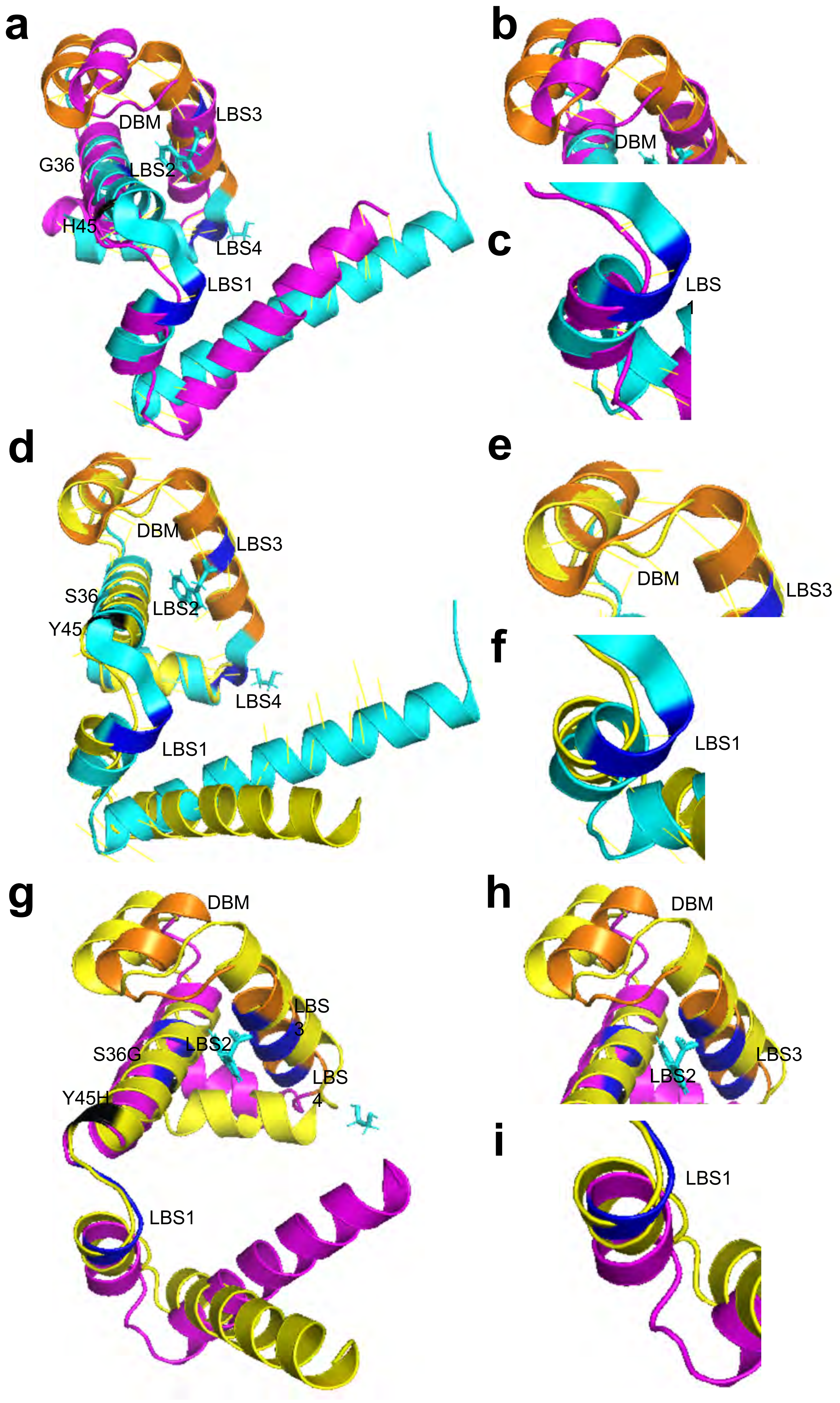
a
DBM
LBS3
G36
LBS2
H45
LBS4
LBS1
b
DBM
c
LBS
1
d
DBM
LBS3
S36
LBS2
Y45
LBS4
LBS1
e
DBM
LBS3
f
LBS1
g
DBM
LBS
3
S36G
LBS2
LBS
4
Y45H
LBS1
h
DBM
LBS3
LBS2
i
LBS1

Supplementary Fig 4. TrpR 3D predicted structures of *Ct* clinical J_SF5 and reference J_UW-36 strains (A-I). (A) TrpR structure of *Ct* mutant strain J_SF5 (magenta) superimposed on the template 6eniA (cyan), with DBM in orange. The LBS 1, 2, 3 and 4 are shown in dark blue, and the aa substitution in relation to J_UW-36 at G36 and H45 is in black. (B and C) Structural changes in DBM and LBS1, respectively, of the mutant are shown. (D) TrpR structure of J_UW-36 (yellow) superimposed on the template 6eniA (cyan) with annotations as per A, B and C. (E and F) Structural changes in DBM and LBS1 of J_UW-36. (G) TrpR 3D predicted structures of J_SF5 superimposed on J_UW-36 with structural changes noted in relation to J_UW-36 at LBS1 and DBM based on S36G and Y45H aa substitution (H and I).
