# Supplementary material for: Tryptophan Operon Diversity Reveals Evolutionary Trends among Geographically Disparate Chlamydia trachomatis Ocular and Urogenital Strains Affecting Tryptophan Repressor and Synthase Function
Source: mBio. 2021 May 11;12(3):e00605-21. doi: 10.1128/mBio.00605-21 (PMC8262981; doi:10.1128/mBio.00605-21)
Supplement: FIG S5 [file mbio.00605-21-sf005.pdf]

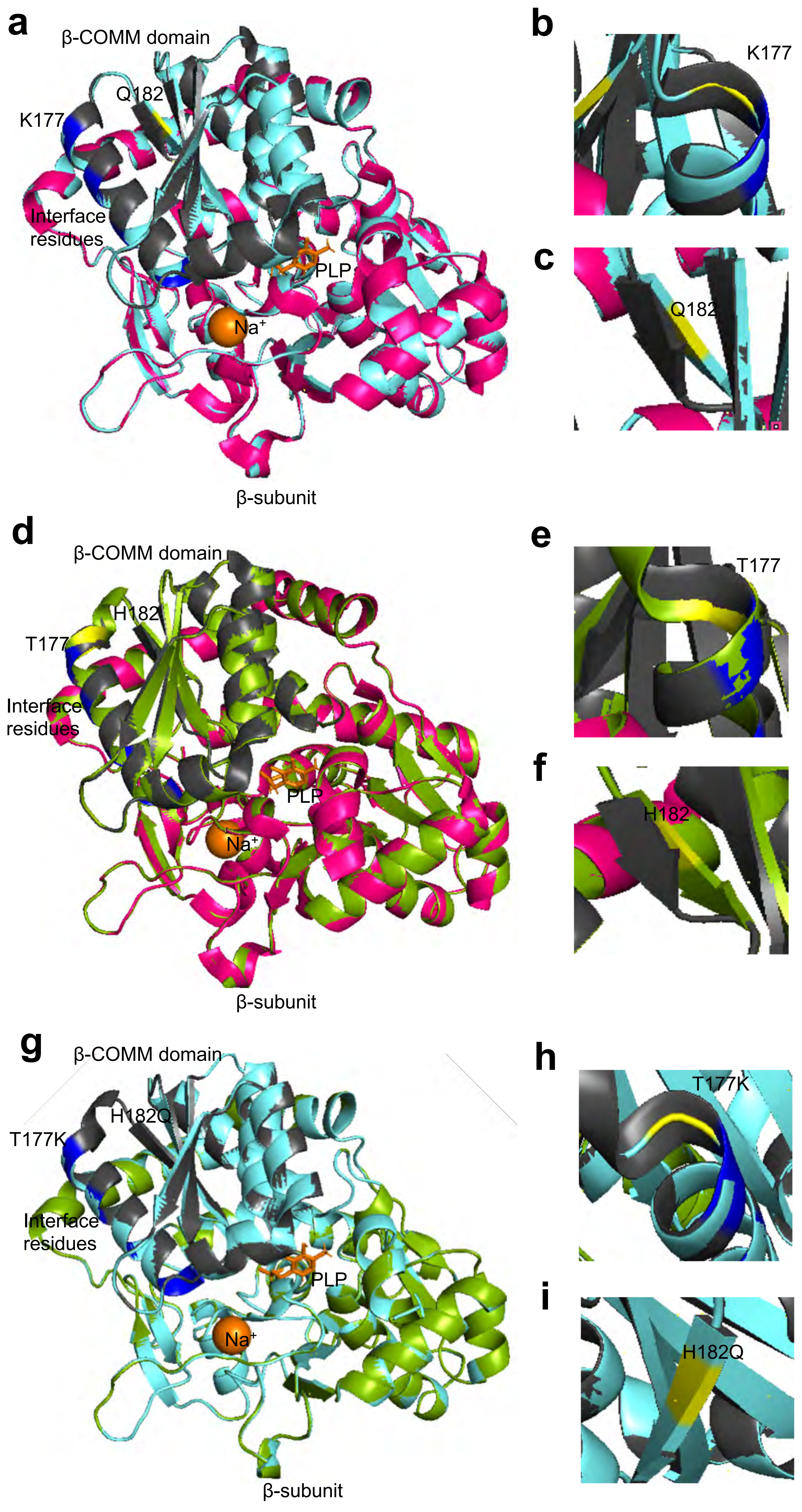
a
β-COMM domain
Q182
K177
Interface residues
PLP
Na+
β-subunit
b
K177
c
Q182
d
β-COMM domain
H182
T177
Interface residues
PLP
Na+
β-subunit
e
T177
f
H182
g
β-COMM domain
H182Q
T177K
Interface residues
PLP
Na+
β-subunit
h
T177K
i
H182Q

Supplementary Fig 5. TrpB 3D predicted structures of *Ct* clinical I_UK913341 and reference I_UW12 strains (A-I). (A) TrpB structure of mutant strain I_UK913341 (cyan) superimposed on the template 5ey5 (magenta), with the COMM domain in charcoal grey. TrpB interface residues that interact with TrpA are shown in blue and the aa substitution in relation to E_Bour at K177 and Q182 are in yellow. (B and C) Structural changes in β subunit interface and COMM domain residues of the mutant E_SotonE8 are shown. (D) TrpB structure of I_UW12 (green) superimposed on the template 5ey5 (magenta) with annotations as per A, B and C. (E and F) Structural changes in β subunit interface and COMM domain residues of the reference I_UW12 are shown. (G) TrpB predicted structures of I_UK913341 superimposed on I_UW12 with structural changes noted in relation to (H) β subunit interface residues on P381L and (I) COMM domain on P304S.
